# Hyaluronan in mesenchymal stromal cell lineage differentiation from human pluripotent stem cells: application in serum free culture

**DOI:** 10.1186/s13287-024-03719-y

**Published:** 2024-05-03

**Authors:** Paul A. De Sousa, Leo Perfect, Jinpei Ye, Kay Samuels, Ewa Piotrowska, Martin Gordon, Ryan Mate, Elsa Abranches, Thomas M. Wishart, David H. Dockrell, Aidan Courtney

**Affiliations:** 1 Centre for Clinical Brain Sciences, University of Edinburgh, Edinburgh, UK; 2 Biotherapeutics and Advanced Therapies, Science Research and Innovation Group, UK Stem Cell Bank, MHRA, South Mimms, UK; 3 Institute of Biomedical Science, Shanxi University, Taiyuan, Shanxi China; 4 Scottish National Blood Transfusion Service, Edinburgh, UK; 5 Department of Molecular Biology, University of Gdansk, Gdańsk, Poland; 6 Roslin Institute, University of Edinburgh, Edinburgh, UK; 7 Centre for Inflammation Research, University of Edinburgh, Edinburgh, UK; 8 Stroma Therapeutics Ltd, Glasgow, UK

**Keywords:** Pluripotent, Human pluripotent stem cells, Mesenchymal stromal cells, Hyaluronan, Cell therapy

## Abstract

**Background:**

Hyaluronan (HA) is an extracellular glycosaminoglycan polysaccharide with widespread roles throughout development and in healthy and neoplastic tissues. In pluripotent stem cell culture it can support both stem cell renewal and differentiation. However, responses to HA in culture are influenced by interaction with a range of cognate factors and receptors including components of blood serum supplements, which alter results. These may contribute to variation in cell batch production yield and phenotype as well as heighten the risks of adventitious pathogen transmission in the course of cell processing for therapeutic applications.

**Main:**

Here we characterise differentiation of a human embryo/pluripotent stem cell derived Mesenchymal Stromal Cell (hESC/PSC-MSC)-like cell population by culture on a planar surface coated with HA in serum-free media qualified for cell production for therapy. Resulting cells met minimum criteria of the International Society for Cellular Therapy for identification as MSC by expression of. CD90, CD73, CD105, and lack of expression for CD34, CD45, CD14 and HLA-II. They were positive for other MSC associated markers (i.e.CD166, CD56, CD44, HLA 1-A) whilst negative for others (e.g. CD271, CD71, CD146). In vitro co-culture assessment of MSC associated functionality confirmed support of growth of hematopoietic progenitors and inhibition of mitogen activated proliferation of lymphocytes from umbilical cord and adult peripheral blood mononuclear cells, respectively. Co-culture with immortalized THP-1 monocyte derived macrophages (Mɸ) concurrently stimulated with lipopolysaccharide as a pro-inflammatory stimulus, resulted in a dose dependent increase in pro-inflammatory IL6 but negligible effect on TNFα. To further investigate these functionalities, a bulk cell RNA sequence comparison with adult human bone marrow derived MSC and hESC substantiated a distinctive genetic signature more proximate to the former.

**Conclusion:**

Cultivation of human pluripotent stem cells on a planar substrate of HA in serum-free culture media systems is sufficient to yield a distinctive developmental mesenchymal stromal cell lineage with potential to modify the function of haematopoietic lineages in therapeutic applications.

**Supplementary Information:**

The online version contains supplementary material available at 10.1186/s13287-024-03719-y.

## Introduction

Hyaluronan (also known as Hyaluronate or Hyaluronic Acid; HA) is a broadly distributed glycosaminoglycan (GAG) polysaccharide, comprised of repeating disaccharides of glucuronic acid and N-acetylglucosamine monomers. Extracellularly, its biophysical properties can manifest as a porous viscoelastic mesh-like structure vital to the physiological function of adult tissue such as cartilage and vitreous humour. This porosity also creates the space necessary for cell migration during embryogenesis, organogenesis, wound repair, tumour metastasis and immune defence. It also functions as a micro environmental cue within tissue niches that co-regulates cell behaviour in these contexts the manner of which depends on its size and availability of cognate factors and receptors (reviewed by [1]).

HA is synthesized by synthases embedded in the inner leaflet of surface plasma membranes and deposited in the extracellular space, after which it can be internalised and distributed within intracytoplasmic and nuclear compartments. It is cleaved by hyaluronidases resulting in availability in biological fluids and tissues as a spectrum of high molecular weight species (1000–8000 kDA) to smaller molecular weight fragments (< 200 kDa). It is not modified by sulfate groups and is not naturally covalently bound to core proteins as for proteoglycans [2]. HA modulation of cell behaviour is mediated by binding to specific hyaluronan binding protein receptors (ie. hyaladhereins) on cell surfaces or within the extracellular matrix (ECM). These include CD44, LYVE-1, HARE, Layilin, RHAMM and TLR4 on the cell surface and aggrecan in the ECM. Binding transduces a range of intracellular signals capable of influencing cell proliferation, energy production, survival, motility, drug resistance and tissue morphogenesis (reviewed in [3]). Tissue specific and conserved effects of HA on stem cell behaviour within niches as well as tissue morphogenesis have been described for haematopoiesis, cardiogenesis, osteogenesis chondrogenesis, neurogenesis and angiogenesis (reviewed in [4–9]). As noted these may depend on HA size. For example, in the developing and adult nervous system low molecular weight HA fragments from endogenous ECM cleaved by hyaluronidases or microbial sources can stimulate defensive response pathways via TLR. Stimulation of same with high molecular weight HA promotes cell proliferation and wound repair [10, 11]. Integral to these diverse outcomes is of course diversity in the repertoire of HA receptors expressed by a cell, as well as enzymes mediating its synthesis and degradation.

In developing mammalian embryos HA is differentially associated with the inner cell mass versus the trophectoderm, and is a prominent feature of post-implantation embryonic cavities and growing tissues [12]. In culture hESC derived from embryo inner cell mass self-renew when encapsulated in 3 Dimensional (3D) Methylacrylated HA hydrogels in first generation feeder cell-conditioned and knockout serum replacement (KOSR) supplemented media as developed by Xu et al. [13, 14]. The same has been reported in 3-D culture of hESC in Tyramine-HA hydrogels in next generation molecular defined serum-free media (mTESR1™; [15]). Sulfated, but not non-sulfated HA can preserve undifferentiated colony phenotype in the short term (3 day) absence of bFGF and feeder conditioned medium in 2 D planar culture [16]. By contrast, we have previously observed that cultivation of hESC on a planar coating of HA in first generation feeder cell conditioned and KOSR supplemented media yielded a proliferative but mortal multi-lineage potent mesenchymal stromal cell (MSC)-like lineage biophysically distinct from hESC and other differentiated cells [17, 18]. Augmentation of this culture system with fetal calf serum also yields an MSC-like lineage and osteogenic potential [19]. Whilst media supplementation with blood serum is common, its usage in the manufacturing of cells for clinical application heightens potential for variation in cell batch production and risk of adventitious pathogen transmission [20]. Here we revisited hESC cultivation on a planar substrate of HA as a route to differentiation of MSC-like cells from pluripotent hESC this time using state of the art serum-free culture systems for hESC and MSC growth appropriate for cell manufacturing for clinical applications. We demonstrate specification of an MSC-like lineage with a molecular identity and potencies to support haematopoietic progenitor growth and adaptive and innate immune cell modulation distinct from adult human bone marrow (hBM)-MSC and hESC. We propose definition of these or other human pluripotent stem cell derived MSC-like cells as Mesenchymal Stromacytes or simply Stromacytes in recognition of their developmental provenance and distinctiveness.

## Methods

### Pluripotent stem cell culture

This study utilised the RC-9 hESC line, suitable as seed material for advanced cell therapies, sourced at passage 29 post derivation [21, 22]. All cell culture was performed in 5% CO_2_ in humidified air (ie. 20% O_2_) at 37 °C. Cryopreservation of undifferentiated hESC and differentiated derivatives was in Cryostor CS10 (Biolife Solution, Washington, USA). hESC growth and differentiation were in multi-well 6 well tissue culture plates (10 cm^2^/well; Cellstar, greiner bio-one, Item No. 657960, Stonehouse, UK), or T25 or T75 flasks (VWR, Leighton, Buzzard, UK). Self-renewal of undifferentiated cells was in StemPro™ hESC SFM on planar coatings of CellStart™ substrate (both from Thermofisher, Paisley UK), used according to manufacturer’s protocols. Spent/fresh media was exchanged daily 6 days a week. Stock cultures were passaged 1:3 by Easy-Cut cell passaging method at 70–100% confluence (EZPassage, Invitrogen by Life Tech, Paisley, UK).

### Differentiation

For differentiation tissue culture plasticware was coated with 0.1mg/ml of hyaluronic acid (HA; 1200 kD, Prod. 385908, Merck-Calbiochem, Nottingham, UK) prepared in Dulbecco’s Phosphate Bufferred Saline (DPBS) and filtered with 0.22µm syringe filter. Coating was for at least 1 h at 4 °C and allowed to reach room temperature before use. Surplus HA solution was aspirated from the wells immediately prior to addition of the cells. HA coating was confirmed by Alcian Blue staining [23].

For RC-9 differentiation from self-renewal conditions comprised of StemPro™ hESC SFM on CellStart, 100% confluent 6 well plates were dissociated with trypLE Select as per manufacturers protocol to obtain single cells. These cells were then passaged onto HA coated plates at 1:1 for three passages after which they were passaged onto CellStart™ and the media switched to StemPro™ MSC SFM (Life Tech, Paisley, UK).

### Other cell culture

Adult bone marrow MSC were used as a reference standard procured commercially from Gibco (StemPro™ BM MSC Cat # A15652) and used between passages 3–5 following cultivation in StemPro™ MSC SFM on CellStart™. Cells were cryopreserved in Cryostor CS10 as for hESC.

### Haematopoietic progenitor growth assay

Fresh UCB-derived MNC fractions were diluted in Stemline II haematopoietic medium (Sigma Aldrich) containing G-CSF (100ng/ml; Peprotech), SCF (100ng/ml; source), TPO (100ng/ml; source), and Flt 3 ligand (50ng/ml; Life Technologies) and plated over pre-established confluent hESC-MSC in T25 flasks at the following concentrations: 0 (hESC-MP feeder only control), 1 × 10^4^, 1 × 10^5^, 2.5 × 10^5^, 5 × 10^5^ and 1 × 10^6^ MNC per flask. For the MNC-only control MNC were plated at 1 × 10^6^ MNC per flask in T25 without hESC-MSC. Flasks were incubated and maintained at 37 °C, 5% CO_2_ in humidified air without media changes. After 7 days of culture, non-adherent cells (ie, MNC fraction containing haematopoietic progenitor/stem cells) were harvested by aspiration of media and subsequent centrifugation at 200× *g* for 5 min. Pelleted cells were subsequently used for flow cytometric analyses, total nuclear counts (TNC) and colony forming unit (CFU) assays. TNC were determined with the ViaCount^®^ assay on the Guava easyCyte system (Millipore) according to manufacturer’s instructions. For the CFU assays, cells were diluted in methylcellulose medium (Methocult, StemCell Technologies) according to manufacturer’s instructions using a dissecting microscope and dark field illumination colonies of ≥ 50 cells were characterised and counted. Photographs of Individual colonies were obtained using an Axiocam (Zeiss) and Axiovision software.

### Mixed peripheral blood mononuclear cell proliferation assay

The blood mononuclear cell modulatory function of MSCs was assessed in an inhibition of proliferation assay in co-culture with cell tracker dye labelled, mitogen stimulated peripheral blood mono-nuclear cells (PBMNCs). One day prior to addition of PBMNCs doubling dilutions of MSCs were titrated in triplicate in 24 well plate at 2.5 × 10^5^ cells to 0.3125 × 10^5^ cells/well in 0.5ml StemMacs medium (SM, Miltenyi Biotec).

PBMNCs were isolated from fresh buffy coats (volume reduced donated units of whole blood obtained from the Scottish National Blood Transfusion Unit) by density gradient separation using Leucosep tubes (Greiner, UK) containing 15ml Ficoll-Paque (GE healthcare, UK). Tubes were centrifuged at 1000 g for 1 min so that ficoll was below the LeucoSep filter which prevents mixing of blood and Ficoll-Paque. Blood was diluted 1:2 with PBS before 30 ml aliquots were pipetted into prepared leucosep tubes and centrifuged at 450 g for 40 min (acceleration/deceleration at 5). The supernatant volume was reduced to 10 ml by pipetting and isolated leucocytes collected by pouring into fresh 50 ml tubes, washed once by centrifugation for 7 min at 350 g. Remaining red blood cells were removed by lysis. The leucocyte pellet was resuspended in 10 ml RBC lysis buffer (BioLegend), incubated for 3 min at room temperature and topped up with PBS before centrifugation at 350 g for 7 min. The wash step was repeated and the leucocyte pellet was resuspended in 10 ml PBS for counting on a haematology cell counter (Sysmex). 2 × 108 leucocytes in10ml PBS (2 × 10^7^/ml) were labelled with an equal volume of eF450 cell tracker dye at 100 nM (Thermofisher) for 20 m at 37 °C, topped up with TexMACS medium (TM, Miltenyi Biotec) and incubated at 4 °C for 20 min. Cells were then washed twice in 50 ml TM and resuspended at 1 × 10^8^/ml in TM.

Labelled PBMC were diluted to 1 × 10^6^/ml in TM supplemented 50 IU/ml IL-2 (Peprotech) for addition of 0.5 ml to wells with pre-plated MSCs to give final PBMC: MSC ratio of 2:1, 4:1, 8:1, 16:1. Proliferation was stimulated by addition of the mitogen PHA (Sigma) at 5 µg/ml Negative control wells omitted PHA and positive control wells containing stimulated PBMNCs alone were included to measure minimum and maximum proliferation respectively. After 6 days culture, at 37 °C, 5%CO2 PBMNCs were harvested by aspiration and washing of wells with PBS. After centrifugation for 7m at 350 g cell pellets were resuspended in 200 µl PBS + 0.5% FCS (Sigma). Halving of fluorescence of labelled daughter cells, in sequential proliferation cycles was measured by flow cytometry using a MacsQuant 10 flow cytometer (Miltenyi Biotec) acquiring 100 µl/sample. Data was analysed using FlowJo software (TreeStar). Debris, doublets and dead cells were excluded from analysis based on FSC and SSC characteristics. Inhibition was calculated using in MSC PBMNC co-cultures relative to% undivided cells in unstimulated negative control.

### Immortalised monocyte specification assay

To evaluate potency to modulate innate immune cells, the immortalised monocyte THP-1 cell line was obtained from ATCC (https://www.atcc.org/) and maintained at 2 × 10^5^ cells/ml in RPMI 1640 medium supplemented with 10% Foetal Calf Serum (FCS) and 2 mmol/L L-glutamine. THP-1 cells (2 × 10^5^ cells/ml) were differentiated to unspecified macrophages (Mɸ) using 100 nM Vitamin D3 (VD3, Sigma-Aldrich) or 200 nM phorbol 12-myristate 13-acetate (PMA, Sigma-Aldrich) for 3d. Differentiation of PMA treated cells was enhanced after the initial 3d stimulus by removing the PMA containing media then incubating the cells in fresh RPMI 1640 (10% FCS, 1% L-glutamine) for a further 5d (PMAr) according to Daigneault et al. [24].

To assess cell potencies to modulate inflammatory cytokine secretions THP-1-Mɸ were stimulated with 100 ug/ml Lipopolysaccharide (LPS) concurrently with concurrently con-culture for 24 h followed by collection and quantification of pro-inflammatory cytokines TNFα and IL6 secretion by Enzyme Linked ImmunoAssays (ELISA) by manufacturers protocols (R&D Systems, Minneapolis, MN, USA). In each experiment co-culture treatments and control LPS treated (ctr LPS+) and untreated in RPMI medium (ctr RPMI) were technically replicated in quadruplicate (n = 4) and normalized with respect to each other in the same assay plate. Inter-plate cytokine assay variation in ctr LPS + levels was 1.5-fold for TNFα (~ 2400–3400 pg/ml) and threefold for IL6 (~ 300–900 pg/ml).

### Flow cytometry

Cell phenotype was assessed by flow cytometry using antibodies to CD49a-f, CD29, CD44, CD71, CD73, CD56, CD133, CD13, CD146, CD105, CD90, CD271, CD166, CD34, CD11b, CD45, CD79A, HLA-I and II (Additional file 2: Table S1). Aliquots of 2 × 10^5^ cells in 100 µl were labelled with optimised concentrations of each antibody separately. After 30min incubation at 4°C samples were washed with 3 ml PBS + 0.5% FBS by centrifugation for 7 min. at 350 g. Pellets were resuspended in 200 µl PBS + 0.5%FBS for acquisition of data for at least 5000 events using a MacsQuant10 flow cytometer (Miltenyi Biotec) after exclusion of debris based on FSC and SSC characteristics. Data was analysed using FlowJo software (Treestar). Dead cells and doublet cells were excluded from analysis based on based on FSC and SSC characteristics. Controls (no antibody) were used to set gates for assessment of marker expression.

Phenotyping of UCB-derived MNC was performed according to International Society of Haemotherapy and Graft Engineering (ISHAGE) guidelines [25]. Total UCB and UCB-derived MNC fractions were washed with PBS containing 5% KOSR (Life Technologies), put through a 70μm cell strainer, suspended in the same buffer at ≤ 1 × 10^6^ cells/ml, and stained in the dark at 5 ± 3 °C for at ≥ 15 min with the following antibodies: fluorescein isothiocyanate (FITC) conjugated anti-CD45, phycoerythrin (PE) conjugated anti-CD133/2 and allophycocyanin-conjugated anti-CD34 according to the manufacturer’s instructions (Miltenyi Biotec. A viability stain, 7-Aminoactinomycin D (7AAD; Cambridge Bioscience, was included to identify live versus dead cells. After staining, cells were washed twice in cold PBS containing 5% KOSR and centrifuged at 500 g for 3 min. Cells were acquired and analysed using Guava EasyCyte System (Millipore). For each sample the total number of CD34+, CD133+ and CD34/CD133 double positive cells were calculated within the live (FAAD+) CD45+ (ie, leucocyte) population.

### RNAseq

A bulkRNAseq analysis was performed on total RNA concurrently isolated from hESC, hESC derived MSC-like cells and human bone marrow derived (hBM) MSC in the current and adjacent report (De Sousa et al., submitted). To structure the analysis the current study focuses on the subset of samples comprised of the undifferentiated RC9 hESC line in StemPro™ SF medium/CellstartTM matrix @ p35 post derivation; hESC-MSC like cells @ p19 post initiation of differentiation mediated by HA in StemPro™ MSC SFM; and adult hBM @ p3 in StemPro™ MSC SFM. Each cell type was biologically replicated in quadruplicate samples of 1 × 10^6^ cryostored cells each.

RNA extractions were carried out using the automated Maxwell RSC Instrument and the Maxwell RSC simplyRNA Cells Kit following the manufacturer’s recommendations. RNA concentrations were measured using Qubit RNA High Sensitivity Kit (Invitrogen) and Qubit 4 fluorometer (Invitrogen). RNA integrity was assessed using the 2100 Bioanalyzer (Agilent) with the RNA 6000 Pico kit (Agilent). Library preparation was performed using Illumina the Stranded mRNA kit (Illumina) with TruSeq RNA Single Indexes (Illumina). Preparation was performed as per manufacturer’s instructions. cDNA library concentrations were measured using Qubit dsDNA High Sensitivity Kit (Invitrogen) and Qubit 4 fluorometer (Invitrogen). cDNA library quality was checked using 2100 Bioanalyzer (Agilent) with the DNA 1000 kit (Agilent). Libraries were sequenced on the NextSeq 500 platform at 40 bp paired-end reads (41 × 2 cycles) using NextSeq 500/550 High Output Kit v2.3 (75 cycles). Two sequencing runs were performed to achieve the required read count (more than 25 million reads per sample).

### Informatics

The standardised RNA sequencing analysis pipeline, nf-core/rnaseq v3.3, was used for quality control, alignment and quantification. Details can be found at https://nf-co.re/rnaseq/3.3. In short, quality control was carried out with FASTQC, adapter and quality trimming with TrimGalore, alignment with STAR and quantification with Salmon. Default settings were used except for STAR “seedSeachStartLmax 25” to increase sensitivity of mapping for 40 bp reads, and TrimGalore “–trim_nextseq 20” to set the Phred score threshold at 20. Reads were aligned to the human genome GRCh37. Count files were imported into R for differential expression analysis with DESeq2 using the default Wald test. *P* values were adjusted using the Benjamini-Hochberg (BH) method and the threshold for differential expression was BH-adjusted *P* < 0.05. Gene ontology enrichment and protein–protein interactions analyses were performed using (1) Qlucore Omics Explorer 3.8 (https://qlucore.com/), and ShinyGo 0.77 [26]. Multi- and two-group comparison of RNA sequence variables were performed at False Discovery Rates (FDR, Q) < 0.05 as reported in figures. Analysis were performed in April 2023. ShinyGO algorithm search parameters of interrogation comprised: Species: Human. FDR cutoff: 0.05; # pathways to show: 20; Pathway size minimum: 2; Pathway size maximum: 2000. Other Options: Redundancies removed, Pathways abbreviated. STRING Pathway Parameters: Human. Display to include up to all genes interrogated.

### Statistical analysis

Non-informatic statistical testing of in vitro culture outcomes was performed using Prism H 5.02 software (GraphPad Software Inc.) by One Way ANNOVA followed by two-tailed post-hoc Dunnett testing of significance.

## Results

### Hyaluronan mediates derivation of mesenchymal stromal cell-like phenotype in serum-free culture systems

**Fig. 1:**
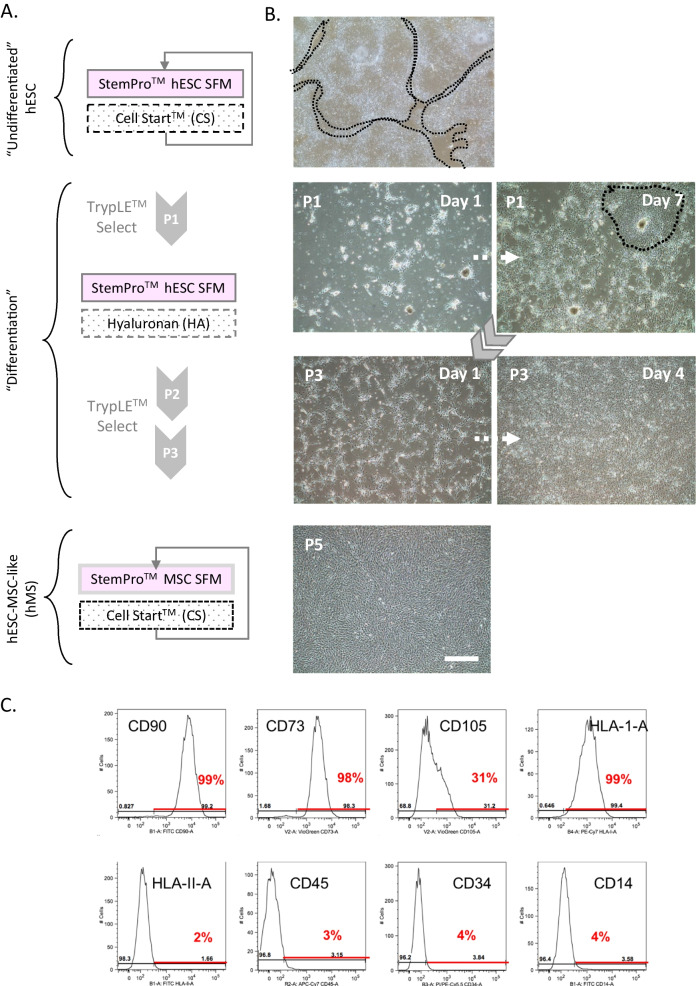
Hyaluronan (HA) mediated differentiation of human mesenchymal stromal cell (MSC)-like cells (aka Mesenchymal Stromacytes; hMS) from human embryo stem cells (hESC). **A** Schematic overview of protocol utilising commercially available serum free media (SFM) systems for hESC and MSC serum free media culture (StemPro™ hESC and MSC, respectively), substrate (CellStart™) and enzyme for cell dissociation (TrypLE™ Select) over successive passages (p). **B** Representative bright field phase contrast microscopy images of cultured cells at passage number and Days post passage. Arrows denote temporal sequence. Dashed lines denote borders of undifferentiated cell colonies. Bar equals 200 µm. **C** Flow cytometry of hMS @ p19 post initiation of differentiation for MSC associated CD90, 73, 105 and HLA-I-A, and non-associated HLA-II-A, CD45, 34 and 14. Percentage of cells positive for each marker in respect of gate set for negative controls

We have previously described the differentiation of an MSC-like lineage utilising Hyaluronan as a planar substrate using the pluripotent H1 and H9 hESC lines [17, 18]. This was presented in a mouse fibroblast feeder cell conditioned and KOSR supplemented media. We began this study with a pilot assessment to verify this outcome on the RC9 hESC line in a human fibroblast conditioned medium. Over the course of three passages using enzymatic methods of cell dissociation, cultures became enriched with a bi-polar fibroblastic cell morphology which by flow cytometry were positive for common MSC associated markers CD146, CD105 and CD90 (~ 40, 95 and 80%, respectively), and negative for the haematopoietic cell marker CD45 (Additional file 1: Fig. S1). Although the RC9 hESC line was originally derived on mitotically inactivated human fibroblast feeders it was transitioned soon after into cell therapy grade serum- and xeno-free medium (StemPro™ hESC SFM) and matrix (CellStart™) for self-renewal [21]. We thus next assessed HA substrate mediated differentiation directly from this culture system into a complementary system for serum- and xeno-free MSC culture (Fig. 1A). Commencing from confluent cultures of the RC9 hESC line under self-renewal conditions, three successive passages of enzymatic dissociation and plating of single cells also yielded a comparable progressive loss of undifferentiated cell colonies and enrichment of a bipolar fibroblastic morphology (Fig. 1B). As with the original method we observed proliferation of these cells in excess of 20 passages in a serum- and xeno-free media designed for cultivation of MSC before retardation of growth became evident practically (data not shown).

To characterise hESC-MSC-like cells, here forth for brevity referred in results as human Mesenchymal Stromacytes (hMS) or simply Stromacytes, we focused on late passage (p) 19 post initiation of differentiation. Independent batches of these were expanded from a cryostored working bank of cells at p15/16 post HA. We first assessed expression of cell surface markers of MSC identity by flow cytometry on two independently generated batches of cells from this working bank. Batches were broadly consistent in forward and side scatter (FSC, SSC) which reflected a greater range in cell diameter and volume (FSC) than cell complexity (SSC) that was low (Additional file 1: Fig. S2), Cells expressed a range of markers commonly associated with adult tissue derived MSC from various sources (eg. Bone, adipose, placental). In descending order of abundance and consistency between batches these were: CD44, CD13, and HLA-I (> 95%); CD166, CD90 and CD73 (70–100%); CD56 (30–40%); and CD105 (15–75%). Cells were predominantly negative for other adult tissue derived MSC-associated markers, namely CD71 and CD271 (~ 5–10%) and CD146 (~ 1–10%). They were also negative for markers not associated with MSC, namely HLA-II and other haematopoietic progenitor lineage markers such as CD34, CD45, CD14 and CD133. Assessment of integrin subunit expression revealed in descending order of abundance and consistency between batches: Integrin alpha 2 and 5 (~ 70–100%); 3 (50–90%); 1, 6 and beta 1 (~ 25–65%) (Fig. 1C, Additional file 1: Figs. S3, S4). On the basis of markers assessed, hMS identify as MSC according to ISCT minimal essential criterion of expression of CD73, 105 and 90 and absence of CD34, CD45 and CD14, the latter denoting an absence of haematopoietic lineages. Expression/absence of other MSC associated markers and integrin subunit profiles flag the likely distinctiveness from primary tissue derived sources and cell-extracellular matrix interaction potential.

### Mesenchymal stromacytes present MSC-like functional properties in co-culture

**Fig. 2:**
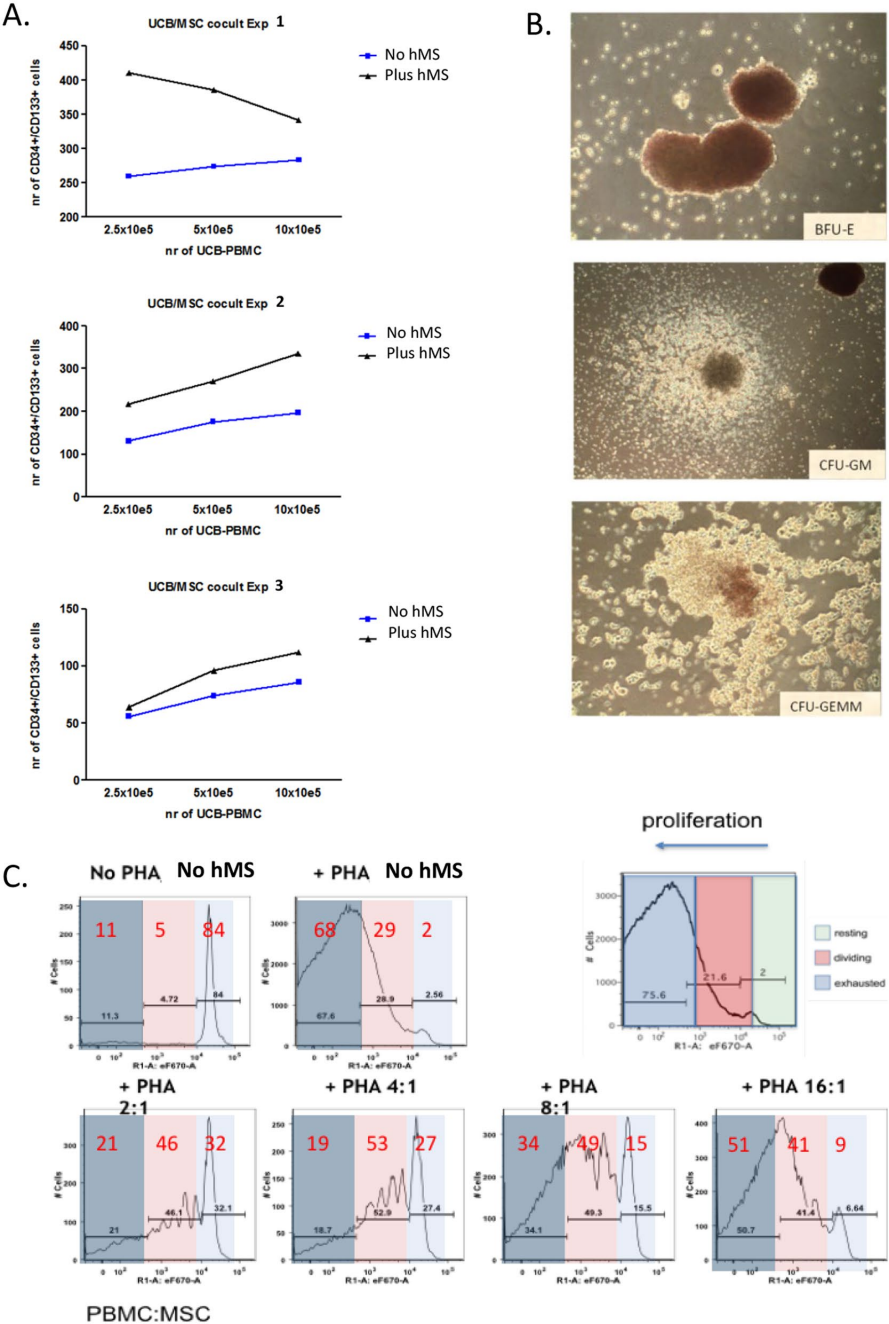
Assessment of potency of hMS co-culture to modulate primary haematopoietic progenitors and lymphocytes. **A** Yields of CD34 and CD133 double positive cells after 7 days of culture with escalating starting number of Umbilical Cord Blood- Peripheral Blood Mononuclear Cells (UCB-PBMC) with or without fixed number of hMS (MSC in figure) in defined haematopoietic progenitor medium. hMS (1 × 10^6^ cells) were co-cultured with 2.5 × 10^5^, 5 × 10^5^ or 10 × 10^5^ cells UCB-PBN for 7 days in culture, at the end of which the number of CD34+/CD133+ cells within the CD45+PBMC population was determined by flow cytometry in 3 independent replications of the experimental design (exp). A total of 20,000, 15,000 and 5000 events were acquired for exp 1 (top), 2 (middle), and 3 (bottom), and absolute numbers of CD34+/CD133+ cells in the latter two exp were normalised to numbers in exp 1. The CD34+/CD133+ cells were quantified firstly by gating on CD45+ cells, and then on 7-Aminoactinomycin (7AAD)+ cells. **B** Qualitative phase contrast microscopy evidence of subsequent differentiation potency of UCB-PBMC following co-culture with hMS for Burst Forming Units of erythroid colonies (BFU-E); progression to late stage colony units of granulocyte–macrophage progenitors (CFU-GM) and granulocyte, erythrocyte, monocyte, and megakaryocytes (CFU-GEEM). **C** Co-culture of hMS with increasing ratio of mitogen (Phytohemagglutinin, PHA) activated adult peripheral blood mononuclear cells (PBMC) inhibits their growth in a ratio dependent manner (**C**). Shown is a representative outcome of two independently replicated experiment. Resting (Green/Blue), Dividing (Red) and Exhausted (Grey) cell subpopulations based on fluorescent cell tracker intensity

MSC are valued for their potency to mediate tissue repair through cell–cell contact and soluble paracrine effects on haematopoietic and tissue resident cells [27]. We thus next investigated functional potencies of hMS to support growth and modulate behaviour of haematopoietic lineages in co-culture. We first considered their capacity to support in vitro expansion of umbilical cord blood derived haematopoietic progenitors, namely CD34 and CD133 double positive mononuclear cells as has been shown for human bone marrow (hBM) derived MSC [28]. Specifically we evaluated yield of these after seven days of co-culture with three independently replicated batches of hMS at p19 post HA differentiation. Each of these was cultured with 3 escalating doses of independent batches of Umbilical Cord Blood-Peripheral Blood Mononuclear Cells (UCB-PBMC) (Additional file 1: Fig. S5A). UCB PBMC co-cultured with hMS consistently yielded more CD34/CD133+ cells than in the absence hMS. These retained differentiation potential to form Burst Forming Units of erythroid colonies (BFU-E), and progression to late-stage colony units of granulocyte–macrophage progenitors (CFU-GM) and granulocyte, erythrocyte, monocyte, and megakaryocytes (CFU-GEEM) (Shown for co-culture Fig. 2B). Merger of differentiated colonies precluded quantification of differentiated progenitor yields. In 2/3 of experiments, yield was directly proportional to UCB PBMC starting number (Fig. 2A, B).

To investigate hMS ability to modulate immune cell responses we assessed capacity to inhibit mitogen activated proliferation of peripheral blood mononuclear cells (PBMC) in co-culture ([29]; Additional file 1: Fig. S5). In the absence of hMS, addition of PHA increased the proportion of maximally proliferated cells from approximately 10% (without PHA treatment) to 70%. This increase in proliferation was reduced when PBMC were co-cultured in the presence of hMS with stronger effects observed at the higher hMS ratios confirming hMS aability to inhibit PBMC proliferation (Fig. 2C representative outcome).

**Fig. 3:**
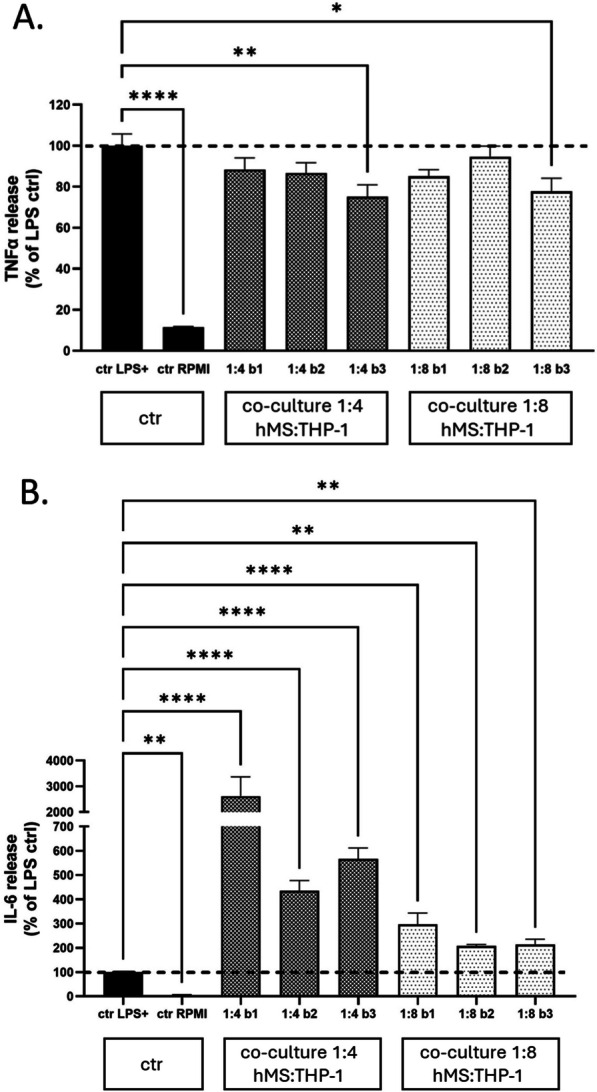
Assessment of potency of hMS co-culture to modulate inflammatory cytokine secretion from LPS stimulated THP-1-Mɸ. Specifically, levels of soluble TNFα (**A**) and IL6 (**B**) in culture media were quantified by ELISAs 24 h after LPS stimulation. Three independent batches of hMS @ p19 post HA (b1, b2, b3) were co-cultured 1:4 and 1:8 with THP-1-Mɸ. In each experiment co-culture treatments and control LPS treated (ctr LPS+) and untreated in RPMI medium (ctr RPMI) were technically replicated in quadruplicate (n = 4) on the same assay plate, with cytokine levels normalized to ctr LPS+ controls, the mean of which set at 100%. Graphs depict ctr LPS+ normalized percentage mean ± standard error on the mean for each cytokine. Statistical significance of difference of level of each cytokine for each hMS batch co-culture treatment versus ctr LPS+ control was determined by one way ANNOVA and post-hoc two tailed Dunnett’s multiple comparison tests. Level of significance: * < 0.05; ** < 0.01; **** < 0.0001

Finally to investigate hMS co-culture ability to modulate innate immune cell responses we used an immortalised THP-1 monocyte as a source of model macrophages (Mɸ) [24] from which pro- to anti-inflammatory subtypes (ie. M1/M2 variants, respectively) can be specified following stimulation with an inflammatory stimulus (lipopolysaccharide, LPS, concurrent with modulating treatments. In this assay, we quantified levels of inflammatory cytokines TNFα and IL6 in culture medium 24 h after LPS stimulation +/− hMS co-culture, these cytokines serving as a surrogate measure of inflammatory status (Additional file 1: Fig. S5c). Three independent batches of hMS @p19 post HA differentiation were evaluated at hMS:THP-1 Mɸ ratios of 1:4 and 1:8 with THP-1. In the absence of cell co-culture, LPS treatment increased soluble TNFα and IL6 levels over tenfold. As compared with LPS treatment alone, hMS co-culture had no (2 batches) or a minor (1 batch) significant reduction in TNFα at both cell co-culture ratios. In contrast, co-culture significantly increased IL-6 levels at both ratios for all 3 batches (Fig. 3).

### RNA phenotyping of mesenchymal stromacyte identity

**Fig. 4:**
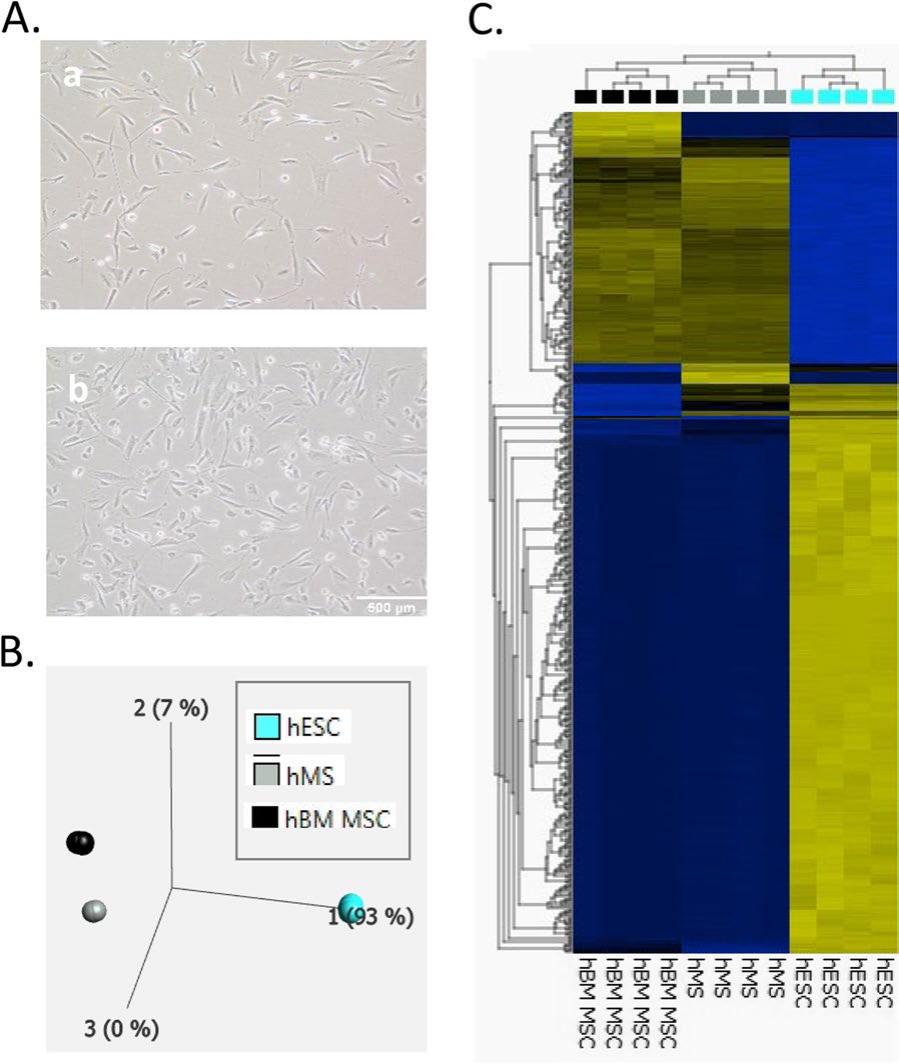
Comparative gross cell morphology and RNA expression of hMS versus hBM-MSC and hESC. **A** Brightfield phase contrast microscopy of hBM-MSC @ p3 (**a**) versus hMS @ p19 post HA (**b**). **B** Principal component analysis and corresponding Heat Map **C** of top 500 most significant differentially expressed genes assessed by bulk cell RNAseq for aforementioned and source RC9 hESC in Stempro™-hESC SF medium on Cellstart™. Significance of *p* ≤ 3.7379e − 18 and False Discovery Rate (FDR) of q ≤ 4.2e−16

Given the similarities between hMS and hBM-MSC in morphology (Fig. 4A), surface marker expression and potencies to modulate haematopoietic lineages we next compared these and source hESC samples by bulk RNAseq. In excess of 196,000 RNA sequence variables associated with a total of 20,964 genes were assessed. Paired comparisons of total gene expression of hMS with both hESC and hBM-MSC manifest comparable ranges of fold changes although there was less difference and significance in the latter comparison (Additional file 1: Fig. S6). Similarity with hBM-MSC and greater distance from hESC was further reinforced by principle component analysis and heat map profiling of the top 500 differentially expressed transcripts (Fig. 4B, C).

**Fig. 5:**
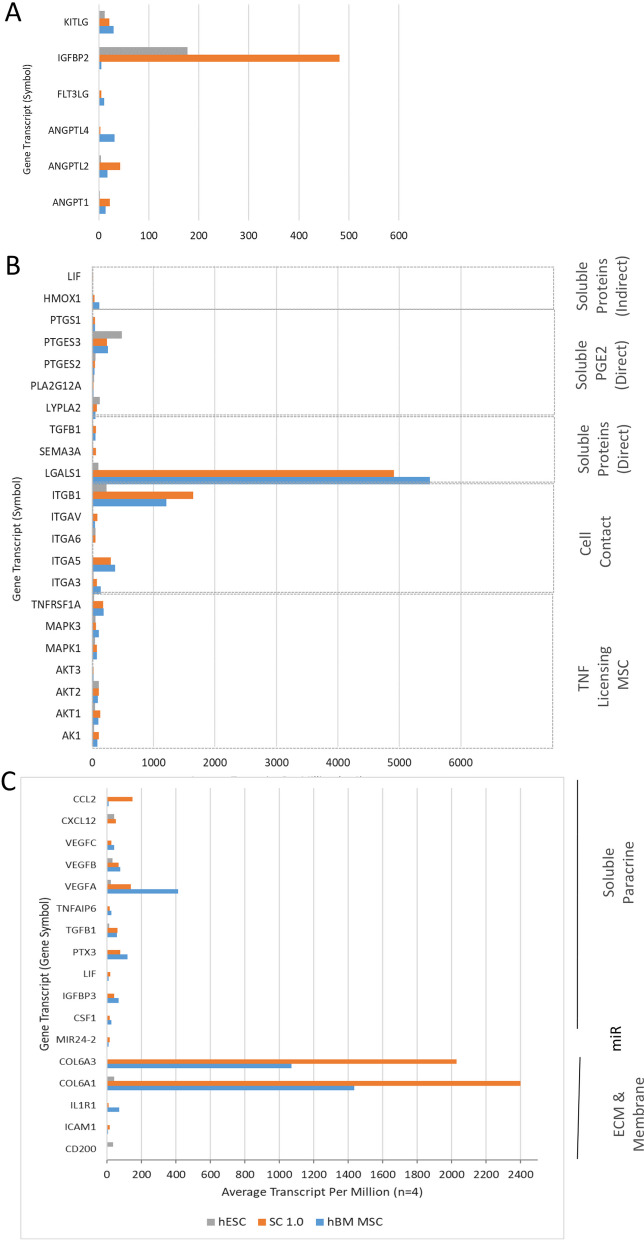
Assessment of expression of selection of gene transcripts associated with MSC support of haematopoietic progenitor expansion (**A**), and adaptive immune T-cell suppression (**B**) and innate immune macrophage specification (**C**). BulkRNAseq of hBM-MSC, hESC, and hMS (in figure referred to as Stromacyte 1.0, SC1.0) assessed in excess of 196,000 RNA sequence variables identified by ENSEMBL IDs that associated with a total of 20,964 genes identified by gene symbols shown in figure. Shown are Average RNA Transcript per million for selected genes for each cell type (from n = 4 biological replicates for each) grouped in **B**, **C** according to form and mode of action. Gene names attributed to each symbol are: KTLG, KIT Ligand; IGFBP2, Insulin Growth Factor Binding Protein 2; FLT3LG, Fms related receptor tyrosine kinase 3 ligand; ANGPTL4, Angiopoietin like 4; ANGPTL2, Angiopoietin like 2; ANGPT1, Angiopoietin 1; LIF, Leukaemia Inhibitory Factor; HMOX1, Heme Oxygenase 1; PTGS1, Prostaglandin-endoperoxide synthase 1; PTGES3 and PTGES2: Prostaglandin E synthase 3 and 2; PLA2G12A, Phospholipase A2 group XIIA; LYPLA2, Lysophospholipase 2; TGFB1, Transforming Growth Factor Beta 1; SEMA3A, Semaphorin 3A; LGALS1, Galectin 1; ITGB1, ITGAV, ITGA6, ITGA5, ITGA3; Integrin subunit beta 1 and alpha V, 6, 5, 3; TNFRSF1A, TNF receptor superfamily member 1A; MAPK3, K1; Mitogen-activated protein kinase 3 and 1; AKT3, 2, 1; AKT serine/threonine kinase 3, 2 and 1; AK1, Adenylate kinase 1; CCL2, C–C motif chemokine ligand 2; CXCL12, C-X-C motif chemokine ligand 12; VEGFC, B, A; Vascular Endothelial Growth Factor C, B, and A; TNFAIP6, TNF alpha induced protein 6; PTX3, Pentraxin 3; IGFBP3, Insulin Growth Factor Binding Protein 3; CSF1; Colony Stimulating Factor 1; miR-24-2, MicroRNA 24-2; COL6A3 and A1, Collagen Type 6 Alpha 3 chaine and Alpha 1 chain; IL1R1, Interleukin 1 receptor type 1; ICAM1, Intercellular adhesion molecule 1; CD200, Cluster of Differentiation 200

We first examined the expression of a selection of 45 genes associated with MSC potency to support haematopoietic progenitor expansion [30], adaptive immune T-cell suppression [31], and innate immune macrophage specification [32]. Genes were selected from BulkRNAseq data on the basis of one or more ENSEMBL ID transcripts associated with a gene being detected in all biological replicates of at least one of the three sample cell types. Transcript expression is presented as Average Number of Transcripts per Million for Gene Symbols to which Ensembl ID relate. Most genes were expressed in all 3 cell types and expression in hMS was comparable or greater to that in hBM-MSC (Fig. 5). Of 6 selected genes associated with support of haematopoietic progenitor expansion (KTLG, FLT3LG, ANGPTL4, ANGPTL2, ANGPT1 and IGFBP2), IGFBP2 expression was the most prominently expressed and in hESC and hMS versus hBM-MSC, respectively (Fig. 5A). Of 22 selected genes involved in MSC suppression of T-cells by indirect soluble proteins (LIF, HMOX1), soluble PGE2 (PTGS1, PTGES3, PTGES2, PLA2G12A, LYPLA2), direct soluble proteins (TGFB1, SEMA3A, LGALS1), cell contact (ITGB1, ITGAV, ITGA6, ITGA5, ITGA3) and TNF licencing (TNFRSF1A, MAPK3, MAPK1, AKT3, AKT2, AKT1, AK1), LGALS1 and ITGB1 were the most prominently expressed and equally so in hMS and hBM-MSC (Fig. 5b). And of genes involved in macrophage specification via soluble paracrine factors (CCL2, CXCL12, VEGFC, B, A, TNFAIP6, TGFB1, PTX3, IGFBP3, CSF1), miR (MIR24-2) and ECM and membrane interactions (COL6A3, COL6A1, IL1R1, ICAM1, aCD200), COL6A3 and 1 were the most prominently expressed and greatest in hMS (Fig. 5C).

**Fig. 6:**
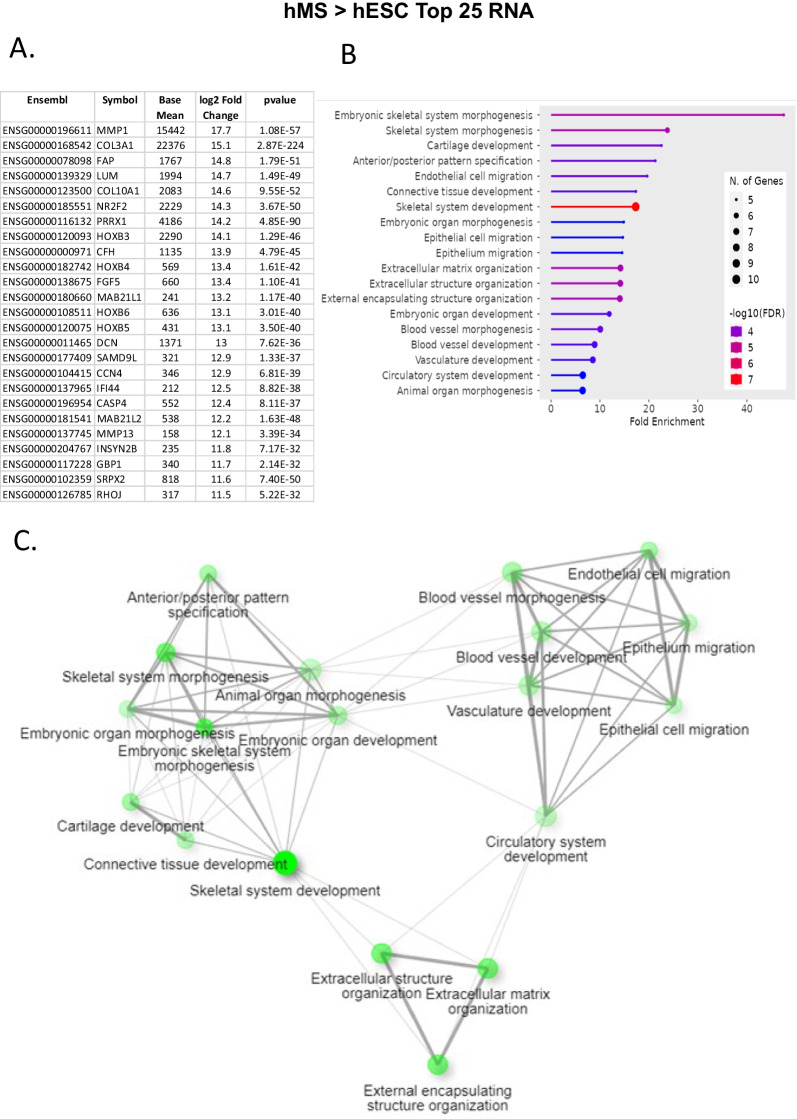
Assessment of top 25 upregulated RNA transcripts in hMS versus hESC. **A** Tabulation of top RNA transcript variant information from left to right: Ensemble ID, Gene Symbol, Bulk RNAseq Base mean value, Log_2_ fold change, and *p* value. **B** ShinyGO 0.77 generated lollipop chart of top 20 Gene Ontologies (vertical axis) in relation to fold enrichment (horizontal axis) interrogating algorithm with top 25 upregulated genes. Legend: number of genes in a GO denoted by circle size and − log_10_FDR of fold enrichment for a GO denoted by colour: blue to red, low to high; FDR, False Discovery Rate. **C** Corresponding GO network graph. Each node represents an enriched GO term, the size of the node corresponds to the number of genes and thickness of lines connecting nodes reflects percent of overlapping genes

**Fig. 7:**
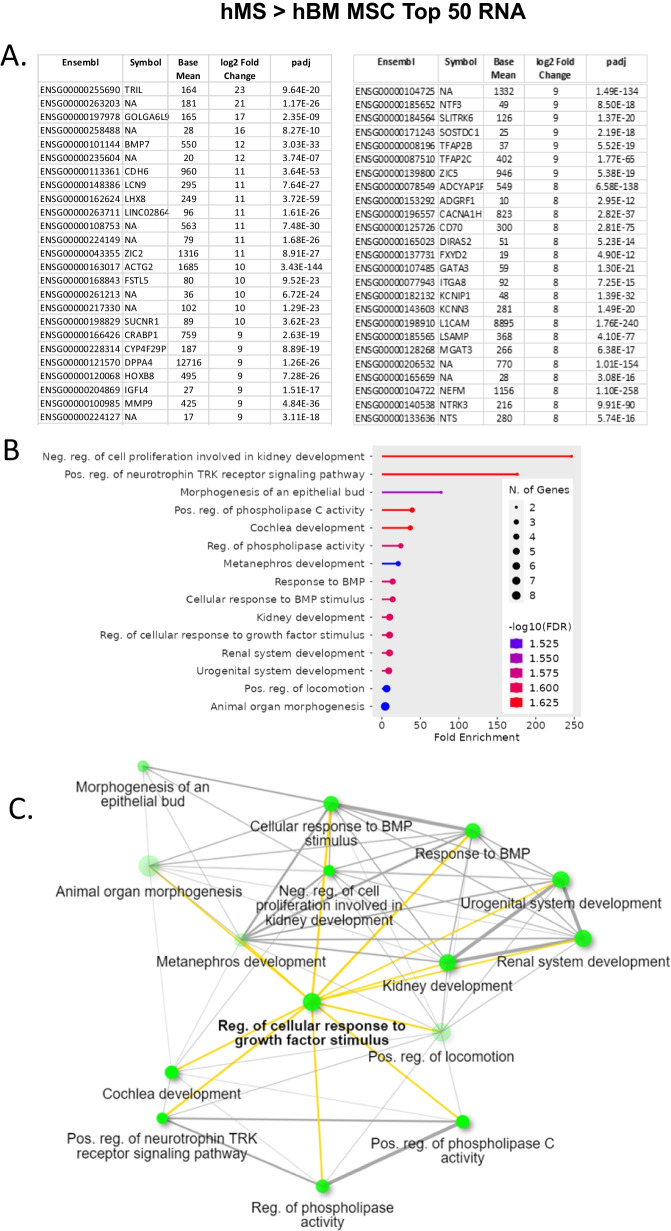
Assessment of top 50 upregulated RNA transcripts in hMS versus hBM-MSC. **A** Tabulation of top RNA transcript variant information from left to right: Ensemble ID, Gene Symbol, Bulk RNAseq Base mean value, Log_2_ fold change, and *p* value. **B** ShinyGO 0.77 generated lollipop chart of top 20 Gene Ontologies (vertical axis) in relation to fold enrichment (horizontal axis) interrogating algorithm with top 50 upregulated genes. Legend: number of genes in a GO denoted by circle size and − log_10_FDR of fold enrichment for a GO denoted by colour: blue to red, low to high. FDR, False Discovery Rate. **C** Corresponding GO network graph. Each node represents an enriched GO term, the size of the node corresponds to the number of genes and thickness of lines connecting nodes reflects percent of overlapping genes. Bolded GO term and yellow highlighted connection reflects centrality of GO

We then carried out gene set enrichment for an unbiased assessment of differences between hMS, hESC and hBM-MSC.We focused on paired comparisons of Gene Ontologies (GO) terms for Biological Process and Curated Reactomes available in the ShinyGO 0.77 platform, the latter chosen to augment understanding of cell differentiation potency and functionality. For each we assessed the top 20 GO terms that were significantly enriched by all genes upregulated and downregulated by more than twofold (Additional file 1: Figs. S7–S10) and subsets of genes upregulated by more than a 100- fold (Figs. 6, 7 and Additional file 1: Figs. S11, S12).

Querying all 4246 transcripts upregulated ≥ twofold in hMS compared with hESC the top 20 Biological Process GO terms manifest as four clusters centred on cell motility and adhesion; vascular development and tissue morphogenesis; skeletal system development; and extracellular matrix organisation. GO terms within these clusters were enriched 2–threefold by 100’s of genes in each (Additional file 1: Figs. S7A, S8A; FDR ≤  − log_10_45). When considering the top 25 RNA transcripts upregulated by 2000-fold or greater, significantly enriched GO terms were essentially the same, now associated in 3 clusters. Each component GO term was enriched in excess of tenfold by 5–10 genes each (Fig. 6; FDR − log_10_4). The top 20 Curated Reactomes for all transcripts upregulated ≥ 2 comprised twelve GO term clusters encompassing interactions, biosynthesis, transport, and signalling of syndecan, collagen, IGF, Receptor Tyrosine Kinase and Interleukins, respectively; cell-ECM interactions; vesicle transport; post-translational protein modification; smooth muscle contraction, blood hemostasis via platelet activation signalling and aggregation; cytokine signalling in the immune system; and neutrophil degradation (Additional file 1: Fig. S11; FDR ≤  − log_10_8). By contrast, querying all 5715 transcripts downregulated ≥ twofold in hMS versus hESC the top 20 GO terms for Biological Process was comprised of seven clusters each enriched 1–threefold by 100’s of genes centred on DNA replication; recombinatorial repair; neurogenesis; ion cation transport; regulation of membrane potential; trans-synaptic signalling; and circulatory system processes (Additional file 1: Fig. S7B, 8B; FDR ≤  − log_10_10). Collectively these may indicate cell acquisition of a mesendodermal phenotype at the expense of undifferentiated processes and ectodermal lineage fate. To identify potential master regulators of hMS identity we interrogated the STRING database [33] for known and predicted protein interactions, for proteins encoded by the top 25 upregulated genes, an arbitrary threshold at which the log fold change in individual genes ranged from 2^11–17^. This predicted 88 interactions substantially exceeding 15 expected randomly. The nexus of this interaction network is Paired Related Homeobox 1 (PRRX1) interacting with other homeobox genes (HOXB3, 4, 5), a transcription factor of an orphan nuclear receptor (NR2F2) and collagen subunits (notably COL3A1) (Additional file 1: Fig. S13, *p* = 0). This nexus suggests a specific developmental and anatomical positioning of cell identity and function for which Collagen is an upstream driver when compared with undifferentiated cells.

### Mesenchymal stromacyte gene expression supports an earlier developmental phenotype when compared to bone marrow MSC

Whereas when compared with hESC, hMS transcriptome reflects a shift away from ectoderm lineage, comparison with hBM-MSC suggests that this progression is incomplete. Querying the 2090 transcripts upregulated ≥ twofold in hMS when compared with hBM-MSC, the top 20 Biological Process GO terms manifest as four clusters centred on cell adhesion; migration, junction organisation; and projection morphogenesis and neuron formation. GO terms within each of these clusters were enriched 2–fivefold by 100’s of genes (Additional file 1: Fig. S9A, S10A; FDR ≤  − log_10_24). A narrowed consideration of GO terms for the top 25 and 50 upregulated genes upregulated by 2^8–ninefold^ yielded a single cluster centred on Regulation of Cellular Response to Growth Factor Stimulus, namely to BMP, Phospholipase C and Neurotrophin-Tyrosine Receptor Kinase receptor signalling pathways and urogenital morphogenesis (Shown for top 50, Fig. 7, 2–8 genes per GO, FDR < 0.03). The top 20 curated Reactomes for all transcripts upregulated ≥ twofold comprised nine GO term clusters encompassing GPCR ligand binding; NCAM interactions; axon guidance; Tyrosine Receptor Kinase signalling; EPH-ephrin mediated cell repulsion; Neuronal system; muscle contraction; and Collagen fibril assembly (Additional file 1: Fig. S12, FDR ≤ -log105). By contrast, the top 20 Biological Process GO terms querying all 2090 transcripts downregulated ≥ twofold in hMS versus hBM-MSC comprised eight clusters each enriched approximately 2–threefold with 40–160 associated genes centred on ossification; cell adhesion; homeostasis; circulatory system processes; urogenital system development; regionalisation; skeletal development; and circulatory system development (Additional file 1: Fig. S9B, S10B, FDR ≤  − log_10_6). Collectively, these results support interpretation of an earlier developmental phenotype for hMS when compared with hBM-MSC. Lastly, to further clarify prospective master regulators of hMS identity and potency we interrogated the STRING database for known and predicted protein interactions with proteins encoded by top 50 upregulated transcripts relative to hBM-MSC. This predicted 37 proteins to have 99 interactions exceeding 40 expected randomly. The nexus of this network is Bone Morphogenic Protein 7, connecting to other hubs centred on Neurotrophic Receptor Tyrosine Kinase 3 (NTRK3) and LIM homeobox 8 (LHX8) (Additional file 1: Fig. S14, *p* = 3.11e−15). These pertain to processes of tissue and cell morphogenesis and differentiation consistent with an earlier developmental phenotype.

## Discussion

Here we report the differentiation of a proliferative MSC-like cell population from pluripotent human embryo stem cells, by virtue of growth on a planar coating of HA in serum-free media systems. These cells have haematopoietic progenitor expansion supportive and adaptive and innate immune cell modulating potencies commonly attributed to adult tissue derived MSCs. In vitro. Additionally, they have a similar molecular identity by cell surface marker flow cytometry and bulk RNAseq. RNA sequence analysis suggests that cells produced by our method are more developmental in their identity.

In vitro studies of functional potency, especially using cell models, such as the THP-1 sourced macrophages used in our study, are surrogate measures of in vivo potency, where effects are niche dependent (reviewed in [34]). Despite this our results support the prospective potency of hMS to modulate primary haematopoietic stem and immune cells. Of particular note as regards modulation of THP-1 macrophage secretion of TNFα and IL6, a comparable outcome was first reported for the capacity of hBM-MSC to modulate an M2 subtype from peripheral blood derived monocytes in the absence of pretreatments to licence hBM-MSC immunomodulatory potency [35].

The need for nomenclature to recognise a more developmentally immature phenotype associated with pluripotent stem cell versus adult tissue derived MSC has been previously argued [36]. Accordingly, we have opted to discriminate mesenchymal stromal cells derived from the former as Mesenchymal Stromacytes or Stromacytes. The latter was first used to reference extracellular matrix proteoglycan depositing corneal stromal cells [37]. It has also been previously applied to reference the mesenchymal progenitor cell sub-population within bone marrow with potency to support the long term growth and differentiation of haematopoitic stem cells in vitro and in vivo [38]. These have been proposed to most closely resemble fetal smooth muscle cells and subendothelial intimal smooth muscle cells; the latter a cell subset with limited development following birth but extensively recruited in atherosclerotic lesions [39]. Our study verified ex vivo support of haematopoietic progenitor expansion. Further, when compared against undifferentiated pluripotent stem cells, gene ontologies enriched by RNA expression in our Stromacytes included for vascular development, smooth muscle contraction, extracellular matrix organisation and signal transduction via the proteoglycan syndecan. Thus, our use of Stromacyte is consistent with prior applications. However, in deference to prior usage of this term and for discrimination from tissue derived cells, our pluripotent stem cell differentiated cells are most likely best described as a *Developmental (Mesenchymal) Stromacyte.* It follows these may vary in relation to differentiation method, particularly recapitulation of presomatic regionalisation, somite development and formation of tissue specific stromal cell populations.

In the last twenty years, an MSC-like identity and functionality has been differentiated from human pluripotent stem cells by different approaches, all of which have reached a similar conclusion of producing a more developmental phenotype (reviewed in Jiang et al. [40]). The first comprised culture based methods, including: (1) co-culture with a murine (OP9) feeder cell [41], (2) embryoid body mediated differentiation [42]; and (3) spontaneous differentiation of adherent cells in cell feeder-free but conditioned culture. All of these methods included supplementation of culture with blood serum [43–47]. A next generation of methods have applied small molecules to manipulate developmental signal transduction events at the heart of mesendoderm versus ectoderm specification (eg. [48, 49]). Specifically, by virtue of modulating the strength, duration and combination of suppression of BMP and TGFβ signal mediators coupled with activation of WNT, it is now possible to differentiate and regionalise presomatic mesoderm, nascent and developed somites and subsequent dermomyotome and sclertome from hPSC [50]. This has offered greater control in the in vitro specification of mesoderm and tissue specific mesenchymal stromal cells. Prior understanding of HA’s role as both instigator and modifier of tissue morphogenesis and cell specification inspired both our and other group explorations of its potential to control pluripotent stem cell behaviour and fate. Like small molecule based methods, our differentiation in serum-free media systems further affirms that differentiation can be achieved without recourse to the complexity of factors serum provides. The proprietary and undisclosed composition of the media used in our study precludes further understanding and dissection of the role and interaction of HA with other cognate factors in the media. However, our results substantiate HA as a determinant of MSC-like fate from pluripotent stem cells.

Planar (2D) cell-adherent culture of human pluripotent stem cells commonly requires either a substrate coating of integrin binding proteins (e.g. fibronectin, laminin, vitronectin) or synthetic surfaces which can mimic what these provide through polymer chemistry and topography [51]. In our original evaluation of HA substrate mediated differentiation in a fibroblast conditioned KOSRT^M^ supplemented medium, and in this study using a serum-free culture media we observed a high degree of non-adherent cell loss in passaging confluent cultures of hESC to HA. This was also seen in the absence of any coating although in this case cells which did adhere failed to proliferate. Differentiating cell growth was observed in this study using HA with a molecular weight (MW) of 1200 kD, whilst the previous observed growth with HA with a MW of 2000 kD, as well as the disaccharide monomer (401.3 D). Thus, one aspect of our approach may involve subculture of hPSC or differentiating derivatives capable of adhering to polystyrene plasticware surfaces with metabolism of HA monomers supporting cell growth, at least when presented in a complex medium such as monomer was tested in.

In development in vivo, HA provides a hydrated matrix thought to facilitate cell proliferation and migration; serve as a reservoir for growth factors that protects them from tryptic digestion; and activate cognate receptors modifying their actions. Embryo inner cell mass and embryonic stem cells derived from them express HA as well as the receptors which mediate their effects. Notable among these is the Homing-Cell Adhesion Molecule (H-CAM, also known as CD44). CD44 is comprised of constitutive (s) and variant (v) exons that can trigger a multitude of different processes from cell proliferation, migration, cell death and survival, depending on the repertoire expressed. Differences between undifferentiated pluripotent and spontaneously differentiating cells in CD44 variant exon expression could constitute another driving factor favouring the selection MSC- like cells at the expense of stem cell renewal. Variation between cells in the expression of HA and CD44s and v exons in the haematopoietic stem cell niche actively controls stem cell honing, quiescence and apoptosis [8], and the expression of CD44v exons plays a role in cancer stem cell renewal and are common prognostic biomarkers of cancer [52].

At the level of CD surface markers associated with adult tissue derived MSC identity, *Developmental Stromacytes* produced in this study lacked CD271 (p75 Low Affinity Nerve Growth Factor Receptor), CD71 (Transferrin Receptor) and CD146 (Melanoma Cell Adhesion Molecule MCAM). CD271 is regarded as an unsuitable universal MSC marker before culture of cells from tissue sources and is inadequate for isolation of MSC from developmental tissues such as umbilical cord blood and Whorton’s jelly [53]. However expression of this surface marker in adipose MSC is associated with higher expression of angiogenic genes and neoangiogenic potential [54]. CD71 is an MSC marker associated with cell proliferation. Its absence is consistent with our assessment of late passage cells [55]. CD146 expression on MSC is associated with their vascular smooth muscle commitment [56].

Potency of our *Developmental Stromacytes* to support and modulate haematopoietic cell lineages was accounted for by assessment of a selection of associated genes. Notable amongst these were IGFBP2; Galectin-1 and Integrin Beta 1; and Collagen VI subunits A3 and A1 with respect to haematopoietic progenitor expansion, suppression of lymphocyte proliferation and macrophage specification, respectively. IGFBP2 supports the survival and cycling of hematopoietic stem cells [57]. Galectins are a class of 15 cell surface and secreted proteins which bind to β-galactoside sugars, such as N-acetyllactosamine. Several members including Galactin-1 have been implicated in tissue MSC potency to modulate adaptive and innate immune cells [58]. Integrin B1 is required for MSC survival and directed migration on collagen and fibronectin substrates in tissue repair [59, 60]. Collagen 6 forms as a unique microfibrillar network between the basement membrane and interstitial matrix of cells and tissues. Congenital mutations in subunits (COL6A1, A2 and A3) cause Ulrich muscular deficiency, a skeletal muscle regeneration deficiency wherein muscle stromal cells support skeletal muscle satellite stem cell renewal and homeostasis through several secreted factors including Collagen VI [61]. Collagen VI has been shown to modulate macrophage activation and cellular functions and other innate and adaptive immune cells directly and indirectly during tissue repair [62]. The cleaved C5 domain of collagen VI alpha 3 chain is a pan-cancer biomarker of poor prognosis and resistance to chemotherapy implicated in tumorigenesis by various mechanisms including sustaining cell stemness, promotion of epithelial to mesenchymal transition, cell migration and angiogenesis [63].

Top Developmental Stromacyte enriched GO terms compared to both hESC and hBM-MSC included cell motility and adhesion consistent with HA associated biological processes and a developmental phenotype. Depending on the comparator this was associated with tissue morphogenesis, notably vascular, urogenital and skeletal (wrt hESC) and cell projection and synapse formation (wrt hBM-MSC). Comparison with the latter reflected a multitude of cellular responses to growth factor stimulus, namely to BMP, Phospholipase C and Neurotrophin-Tyrosine Receptor Kinase receptor signalling. Top differentially expressed genes in either comparison featured HOX (HOXB3, 4, 5 and associated major transcriptional regulators (PRRX1, LIM homeobox18, NR2F2) interacting and likely regulating downstream expression of collagen subunits (COL3A1), BMP7 and Neurotrophic Receptor Tyrosine Kinases (NTRK3). Despite the uniformity of morphology in culture hMSC, cell heterogeneity is likely to have existed which our utilisation of bulk RNA sequencing would not discriminate. Thus, the extent to which these molecules interplay within each other within any given cell is unclear. However, understanding the prominence of expression of these molecules provides the foundation to future work to understand and manipulate cell identity and potencies to differentiate or modulate other cells. For example, homeobox genes specify regions of the body plan of an embryo along the head–tail axis and determine cell fate and tissue patterning during tissue morphogenesis. In mammalian development HOXB3 is expressed in hindbrain rhombomeres that regulate the development of hindbrain cranial and motor neurons. It is also expressed along with HOXB4, and B5 as well as other HOX paralogs along the anterior to posterior axis in developing thymus and lung tissue and proximate skeletal vertebrae [64]. BMP7 promotes both chondrogenic and osteogenic differentiation in MSC [65] and its transgenic overexpression in hBM-MSC improves effectiveness in healing bone fractures as compared with non-transgene expressing cells [66].

Since the first study describing Mesenchymal Stem or Stromal Cells [67] preclinical investigations have substantiated promise of their utility for tissue reparation. Clinical validation of this promise remains a work in progress and challenged by variability of outcomes. Whereas initially transplanted stem cell lineage potency was thought to replace cells or tissue structure lost or damaged following acute injuries or chronic disease, current popular emphasis centers on cell signalling potencies to modulate somatic and immune cell behaviour in healing [68]. MSC derived from formed tissues are now understood to derive from vasculogenic pericytes [69], but in the course of development, pericytes originate from MSC-like mesoderm progenitors [70, 71]. Both MSC and pericytes have demonstrated potency to differentiate to osteogenic, chondrogenic, adipogenic and myogenic cells. Analysis of the multilineage potential of single MSC derived parent and daughter clones suggests a hierarchical schema for MSC self-renewal and differentiation in which a self-renewing multipotent MSC gives rise to more restricted self-renewing progenitors that gradually lose differentiation potential until a state of complete restriction to the fibroblast is reached [72]. It is likely that with lineage restriction there are concurrent changes in cell–cell signalling potency over and above differences associated with tissue of origin or in vitro differentiation from pluripotent stem cells. Recently, multi-omic analysis of MSC demonstrate that cell ageing alters immunomodulatory activity [73].

## Conclusions

In conclusion we report here that cultivation of human pluripotent stem cells on a planar substrate of HA in serum-free culture media systems is sufficient to yield a distinctive developmental mesenchymal stromal cell lineage. In vitro assessments of functional potency and molecular assessment of identity by flow cytometry and RNA sequencing reported here substantiate their potential to exhibit tissue reparative potencies as associated with MSC of diverse tissue origins when tested in preclinical models of acute injury or chronic diseases. The utilisation of serum-free culture systems, simplicity of the method, and the proliferative capacity of resulting cell populations all favour scalable manufacture of these cells for therapeutic applications.

### Supplementary Information

**Additional file 1: Figure S1.** Verification of a method of differentiating MSC-like cells from hESC. To assess RC-9 differentiation from self-renewal conditions comprised of Human Dermal Fibroblast Conditioned Medium (HDF-CM) in CellStart^TM^,stock cultures of RC-9 in StemPro^TM^ hESC SFM cells were sequentially transitioned every second media exchange through dilutions prepared with 20, 40, 60, 80 and 100% HDF-CM. HDF-CM was prepared as described by Fletcher et al (2006) based on the method of Xu et al., (2001). From 40% HDF-CM, the EZPassaging method was replaced with collagenase-IV based cell passaging as per aforementioned references for cell self-renewal and differention. After two passages in 100% CM passaging cells 1:3, they were moved to hyaluronan-coated plasticware upon reaching 100% confluence. Cells were detached using collagenase IV solution were re-plated 1:2 on the HA-coated plates. After two passages with collagenase on HA, the cells were passaged with trypLE Select (Gibco by Life Tech, Paisley, UK ) as per manufacturer’s instructions onto CellStart^TM^, until the disappearance of colony-like clusters from the cultures and appearance of a uniform, bi-polar fibrblastic cell morphology. During the trypLE Select passaging, the splitting ratio ranged from 1:1 to 1:6 depending on confluence or transition from growth in 6-well plates to T25 and T75 flasks (VWR, Leighton Buzzard, UK).(I) Brightfield microscopy of cultures of the RC9 hESC line subject to enzymatic dissociation with collagenase (p1 & p2) and TrypleSelect (p3) on a planar substrate of HA in Human Dermal Fibroblast Conditioned Medium (HA/HDF CM) . (II) Flow cytometry characterization of RC9 HA/HDF CM derived MSC-like cells @ passage 6 post transition to a planar coating of HA for CD146, 105, CD90 and CD45. Grey profile, isotype antibody. Red profile, CD epitope targeted antibody. Percentage is proportion of CD epitope targeted cells against gating for isotype control. **Figure S2**. Forward and Side Scatter Flow cytometry profiles for independent assessments hMS @ p19 post HA surface markers. Corresponds to outcomes presented in Fig. 1C, and Suppl. Fig. 3 & 4. **Figure S3.** Flow cytometry profiles for cell surface markers on hMS @ p19 post HA. Independent replicate experiments for assessment of cell surface markers denoted in blue and red as relates to forward/side scatter profiles depicted in Suppl. Fig. 2. Surface markers identified according to Cluster of Differentiation (CD) designation. Correspondence to gene names as follows: CD49 a, b, c, d, e, f, correspond to Integrin alpha 1, 2, 3, 4, 5 & 6; CD29, Integrin beta 1; CD44, Matrix adhesion molecule that adheres to HA, collagen, laminin and fibronectin; CD71 Transferrin Receptor; CD73, 5’Ribonucleotide Phosphohydrolase, also known as ecto-5’ nucleotidase (NT5E); CD56, Neural Cell Adhesion Molecule (NCAM); CD133, Prominin-1; CD13, Alanyl Aminopeptidase N (ANPEP); CD146, Melanoma Cell Adhesion Molecule (MCAM); CD105, Endoglin; CD90, Thy1 Cell Surface Antigen; CD271, p75 Low Affinity Nerve Growth Factor Receptor; CD166, Activated Leukocyte Cell Adhesion Molecule, ALCAM; CD34, Transmembrane Glycoprotein associated with haematopoietic precursors; HLA I & II, Human Leukocyte Antigen Type I & 2. **Figure S4.** Summary of flow cytometry outcomes in two independent batches of hMS @p19 post HA. Production and assessment of batches temporally separated by 5 weeks. Profiles for each outcome depicted in Supplementary Figures 2-3. **Figure S5.** Schematics of experimental designs for assessment of hMS potencies to modulate haematopoietic lineages in cell co-culture. Depicted are for assessment of: (A) haematopoietic progenitor expansion. (B) Inhibition of mitogen (Phytohemagglutinin, PHA) activated adult peripheral blood mononuclear cells (PBMC) proliferation. (C) modulation of soluble pro-inflammatory cytokine TNFα and IL6 secretion from immortalized THP-1-Monocyte (Mɸ). **Figure S6.** Enhanced Volcano depiction of distribution of changes in total gene expression assessed by bulkRNA sequences in paired comparisons: (A) hMS vs hESC. (B) hMS vs hBM-MSC. Vertical axis: Significance as –Log_10_*P*. Horizontal axis: Log_2_ fold change. Circles constitute RNA transcript variant. Legend depicts range of statistical significance of fold change for transcript according to colour: Purple, non-significant. Blue to yellow increasing significance. Dashed lines denote adjusted p-value < 0.05. Total number of genes assessed 20964. **Figure S7.** Chart of Top 20 GO for Biological Processes enriched by RNA transcripts exhibiting ≥ 2 fold changes in hMS vs hESC. ShinyGO v 0.77 Gene Ontology Enrichment Analysis generated lollipop chart of significantly enriched GO following interrogation of algorithm with genes upregulated (A, n=4246) and downregulated (B, n= 5715) ≥ 2 fold in hMS vs hESC. Gene Ontologies (vertical axis) in relation to fold enrichment (horizontal axis). Legend: number of genes in a GO denoted by circle size and –log_10_FDR of fold enrichment for a GO denoted by colour: blue to red, low to high. FDR, False Discovery Rate. Performed 14 April 2023. **Figure S8.** Network of Top 20 GO for Biological Processes enriched by RNA transcripts exhibiting ≥ 2 fold changes in hMS vs hESC. ShinyGO v 0.77 Gene Ontology Enrichment Analysis generated graph of significantly enriched GO e following interrogation of algorithm with genes upregulated (A, n=4246) and downregulated (B, n= 5715) ≥ 2 fold in hMS vs hESC. Network corresponds to chart in Suppl. Fig. 7. Each node represents an enriched GO term, the size of the node corresponds to the number of genes and thickness of lines connecting nodes reflects percent of overlapping genes. Performed 14 April 2023. **Figure S9.** Chart of Top 20 GO for Biological Processes enriched by RNA transcripts exhibiting ≥ 2 fold changes in hMS vs hBM-MSC. ShinyGO v 0.77 Gene Ontology Enrichment Analysis generated graph of significantly enriched GO following interrogation of algorithm with genes upregulated (A, n=2090) and downregulated (B, n=1593) ≥ 2 fold in hMS vs hBM-MSC. Gene Ontologies (vertical axis) in relation to fold enrichment (horizontal axis). Legend: number of genes in a GO denoted by circle size and –log_10_FDR of fold enrichment for a GO denoted by colour: blue to red, low to high. FDR, False Discovery Rate.Performed 14 April 2023. **Figure S10.** Network of Top 20 GO for Biological Processes enriched by RNA transcripts exhibiting ≥ 2 fold changes in hMS vs hBM hESC. ShinyGO v 0.77 Gene Ontology Enrichment Analysis generated graph of significantly enriched GO following interrogation of algorithm with genes upregulated (A, n=2090) and downregulated (B, n=1593) ≥ 2 fold in hMS vs hBM-MSC. Network corresponds to chart in Suppl. Fig 9. Each node represents an enriched GO term, the size of the node corresponds to the number of genes and thickness of lines connecting nodes reflects percent of overlapping genes. Performed 14 April 2023. **Figure S11.** Top 20 Curated Reactome GO enriched by RNA transcripts exhibiting a ≥ 2 fold upregulation in hMS vs hESC. ShinyGO v 0.77 generated chart (A) and corresponding network (B) of significantly enriched GO following interrogation of algorithm with genes upregulated ≥ 2 fold d in hMS vs hESC (n=4246). Chart: Gene Ontologies (vertical axis) in relation to fold enrichment (horizontal axis). Legend: number of genes in a GO denoted by circle size and –log10FDR of fold enrichment for a GO denoted by colour: blue to red, low to high. FDR, False Discovery Rate. Network: Each node represents an enriched GO term, the size of the node corresponds to the number of genes and thickness of lines connecting nodes reflects percent of overlapping genes. **Figure S12.** Top 20 Curated Reactome GO enriched by RNA transcripts exhibiting a ≥ 2 fold upregulation in hMS vs hBM-MSC. ShinyGO v 0.77 generated chart (A) and corresponding network (B) of significantly enriched GO following interrogation of algorithm with genes upregulated ≥ 2 fold d in hMS vs hBM-MSC (n=2090). Chart: Gene Ontologies (vertical axis) in relation to fold enrichment (horizontal axis). Legend: number of genes in a GO denoted by circle size and –log10FDR of fold enrichment for a GO denoted by colour: blue to red, low to high. FDR, False Discovery Rate. Network: Each node represents an enriched GO term, the size of the node corresponds to the number of genes and thickness of lines connecting nodes reflects percent of overlapping genes. **Figure S13.** ShinyGo 0.76 STRING protein-protein Interaction pathway analysis for proteins encoded by top 25 RNA transcript associated genes upregulated in hMS vs hESC denoted in Figure 6. Proteins encoded by RNA transcripts depicted by coloured nodes with associated gene symbol name. Colour serves only as visual aid. Line Connectors depict predicted associations: Red line-indicates presence of fusion evidence; Green line – neighbourhood evidence; Blue line – concurrence evidence; Purple line – experimental evidence; Yellow line – text mining evidence; Light blue line – database evidence; Black line – co-expression evidence. Thickness of line indicates the degree of confidence prediction. Legend: Proteins: Number of proteins depicted in figure. Interactions: Number of predicted known and predicted interactions. Expected interactions: Number of interactions expected by chance. P= significance. **Figure S14.** ShinyGo 0.76 STRING protein-protein Interaction pathway analysis for proteins encoded by top 50 RNA transcript associated genes upregulated in hMS vs hBM-MSC denoted in Figure 7. Proteins encoded by RNA transcripts depicted by coloured nodes with associated gene symbol name. Colour serves only as visual aid. Line Connectors depict predicted associations: Red line-indicates presence of fusion evidence; Green line – neighbourhood evidence; Blue line – concurrence evidence; Purple line – experimental evidence; Yellow line – text mining evidence; Light blue line – database evidence; Black line – co-expression evidence. Thickness of line indicates the degree of confidence prediction. Legend: Proteins: Number of proteins depicted in figure. Interactions: Number of predicted known and predicted interactions. Expected interactions: Number of interactions expected by chance. P= significance reported by algorithm.

**Additional file 2: Table S1.** Flow Cytometry Antibody Information. From left to right columns: Target protein which antibody directed towards, Fluorochrome conjugate, Distributor, Catalog Number, and working dilution. APC, Allophycocyanin; FITC, Fluorescein isothiocyanate; PerCP, Peridinin Chlorophyll Protein Complex; PE, Phycoerythrin.

## Data Availability

The RC9 (RCe013-A) cell line was derived in compliance with EU Tissues and Cells Directives and UK Human Fertilisation and Embryology and Human Tissue Authority licenses warranting ethical procurement and utility to serve as source material for advanced therapies in the EU. It is registered with the EU hPSC^reg®^ (https://hpscreg.eu/cell-line/RCe013-A) and can be procured for evaluation and as source material for product development from the UK National Institute for Biological Standards (NIBSC) UK Stem Cell Bank (UKSB) ( https://www.nibsc.org/ukstemcellbank). The raw datasets generated and analysed during the current study are not publicly available due to commercial sensitivity. Differentially expressed gene lists are available for non-commercial use from the corresponding author under confidentiality agreement on reasonable request.

## Abbreviations

HA: Hyaluronan
hESC: Human embyro stem cells
hBM: Human bone marrow
MSC: Mesenchymal stromal cells
hMS: Mesenchymal stromacytes (stromacytes)
