# Supplementary material for: Hyaluronan in mesenchymal stromal cell lineage differentiation from human pluripotent stem cells: application in serum free culture
Source: Stem Cell Res Ther. 2024 May 3;15:130. doi: 10.1186/s13287-024-03719-y (PMC11069290; doi:10.1186/s13287-024-03719-y)
Supplement: Supplementary file 1 — Additional file 1: Figure S1. Verification of a method of differentiating MSC-like cells from hESC. To assess RC-9 differentiation from self-renewal conditions comprised of Human Dermal Fibroblast Conditioned Medium (HDF-CM) in CellStartTM,stock cultures of RC-9 in StemProTM hESC SFM cells were sequentially transitioned every second media exchange through dilutions prepared with 20, 40, 60, 80 and 100% HDF-CM. HDF-CM was prepared as described by Fletcher et al (2006) based on the method of Xu et al., (2001). From 40% HDF-CM, the EZPassaging method was replaced with collagenase-IV based cell passaging as per aforementioned references for cell self-renewal and differention. After two passages in 100% CM passaging cells 1:3, they were moved to hyaluronan-coated plasticware upon reaching 100% confluence. Cells were detached using collagenase IV solution were re-plated 1:2 on the HA-coated plates. After two passages with collagenase on HA, the cells were passaged with trypLE Select (Gibco by Life Tech, Paisley, UK ) as per manufacturer’s instructions onto CellStartTM, until the disappearance of colony-like clusters from the cultures and appearance of a uniform, bi-polar fibrblastic cell morphology. During the trypLE Select passaging, the splitting ratio ranged from 1:1 to 1:6 depending on confluence or transition from growth in 6-well plates to T25 and T75 flasks (VWR, Leighton Buzzard, UK).(I) Brightfield microscopy of cultures of the RC9 hESC line subject to enzymatic dissociation with collagenase (p1 & p2) and TrypleSelect (p3) on a planar substrate of HA in Human Dermal Fibroblast Conditioned Medium (HA/HDF CM) . (II) Flow cytometry characterization of RC9 HA/HDF CM derived MSC-like cells @ passage 6 post transition to a planar coating of HA for CD146, 105, CD90 and CD45. Grey profile, isotype antibody. Red profile, CD epitope targeted antibody. Percentage is proportion of CD epitope targeted cells against gating for isotype control. Figure S2. Forward and Side Scatt [file 13287_2024_3719_MOESM1_ESM.pptx]

## Slide 1
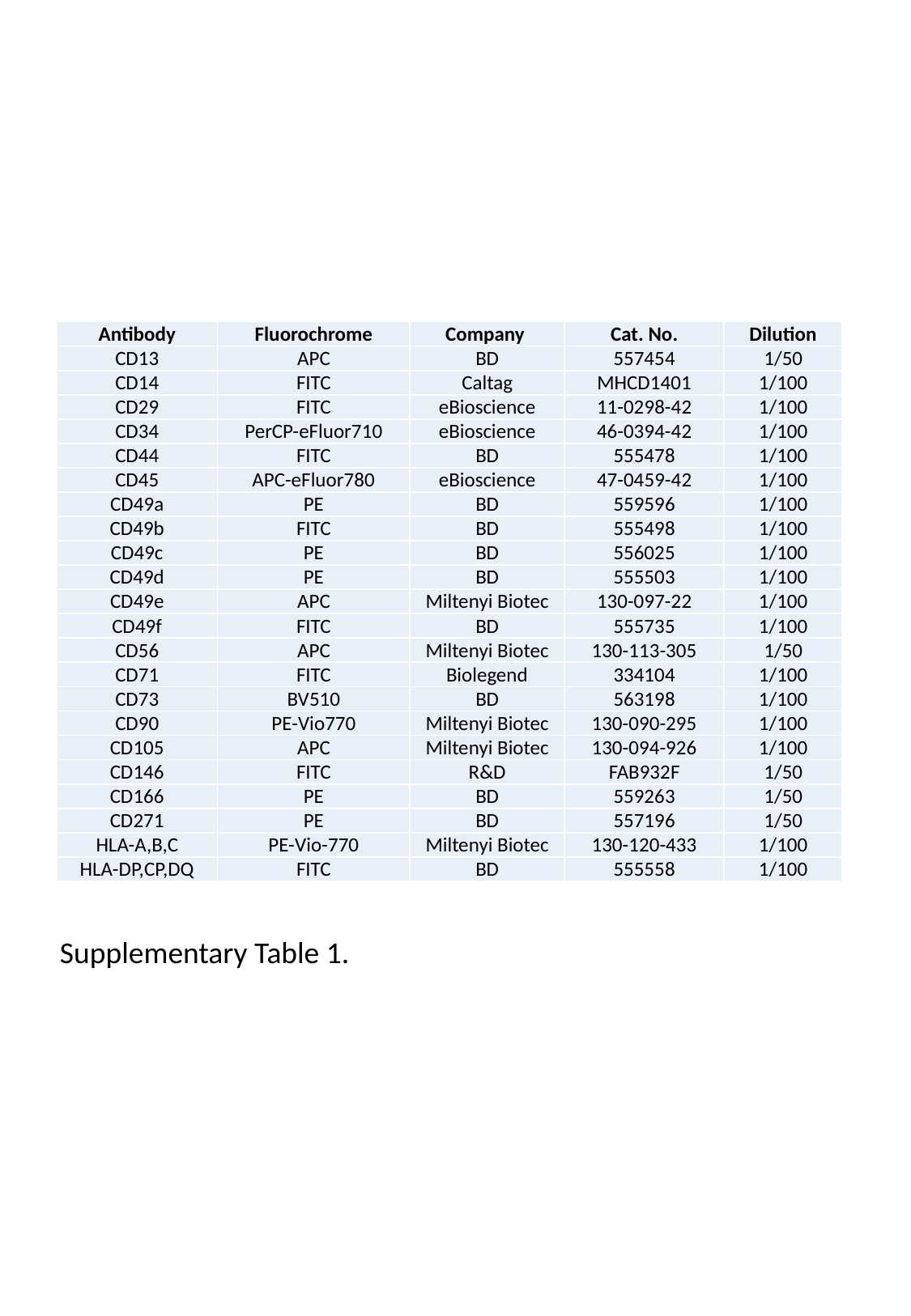

| Antibody | Fluorochrome | Company | Cat. No. | Dilution |
| --- | --- | --- | --- | --- |
| CD13 | APC | BD | 557454 | 1/50 |
| CD14 | FITC | Caltag | MHCD1401 | 1/100 |
| CD29 | FITC | eBioscience | 11-0298-42 | 1/100 |
| CD34 | PerCP-eFluor710 | eBioscience | 46-0394-42 | 1/100 |
| CD44 | FITC | BD | 555478 | 1/100 |
| CD45 | APC-eFluor780 | eBioscience | 47-0459-42 | 1/100 |
| CD49a | PE | BD | 559596 | 1/100 |
| CD49b | FITC | BD | 555498 | 1/100 |
| CD49c | PE | BD | 556025 | 1/100 |
| CD49d | PE | BD | 555503 | 1/100 |
| CD49e | APC | Miltenyi Biotec | 130-097-22 | 1/100 |
| CD49f | FITC | BD | 555735 | 1/100 |
| CD56 | APC | Miltenyi Biotec | 130-113-305 | 1/50 |
| CD71 | FITC | Biolegend | 334104 | 1/100 |
| CD73 | BV510 | BD | 563198 | 1/100 |
| CD90 | PE-Vio770 | Miltenyi Biotec | 130-090-295 | 1/100 |
| CD105 | APC | Miltenyi Biotec | 130-094-926 | 1/100 |
| CD146 | FITC | R&D | FAB932F | 1/50 |
| CD166 | PE | BD | 559263 | 1/50 |
| CD271 | PE | BD | 557196 | 1/50 |
| HLA-A,B,C | PE-Vio-770 | Miltenyi Biotec | 130-120-433 | 1/100 |
| HLA-DP,CP,DQ | FITC | BD | 555558 | 1/100 |
Supplementary Table 1.

## Slide 2
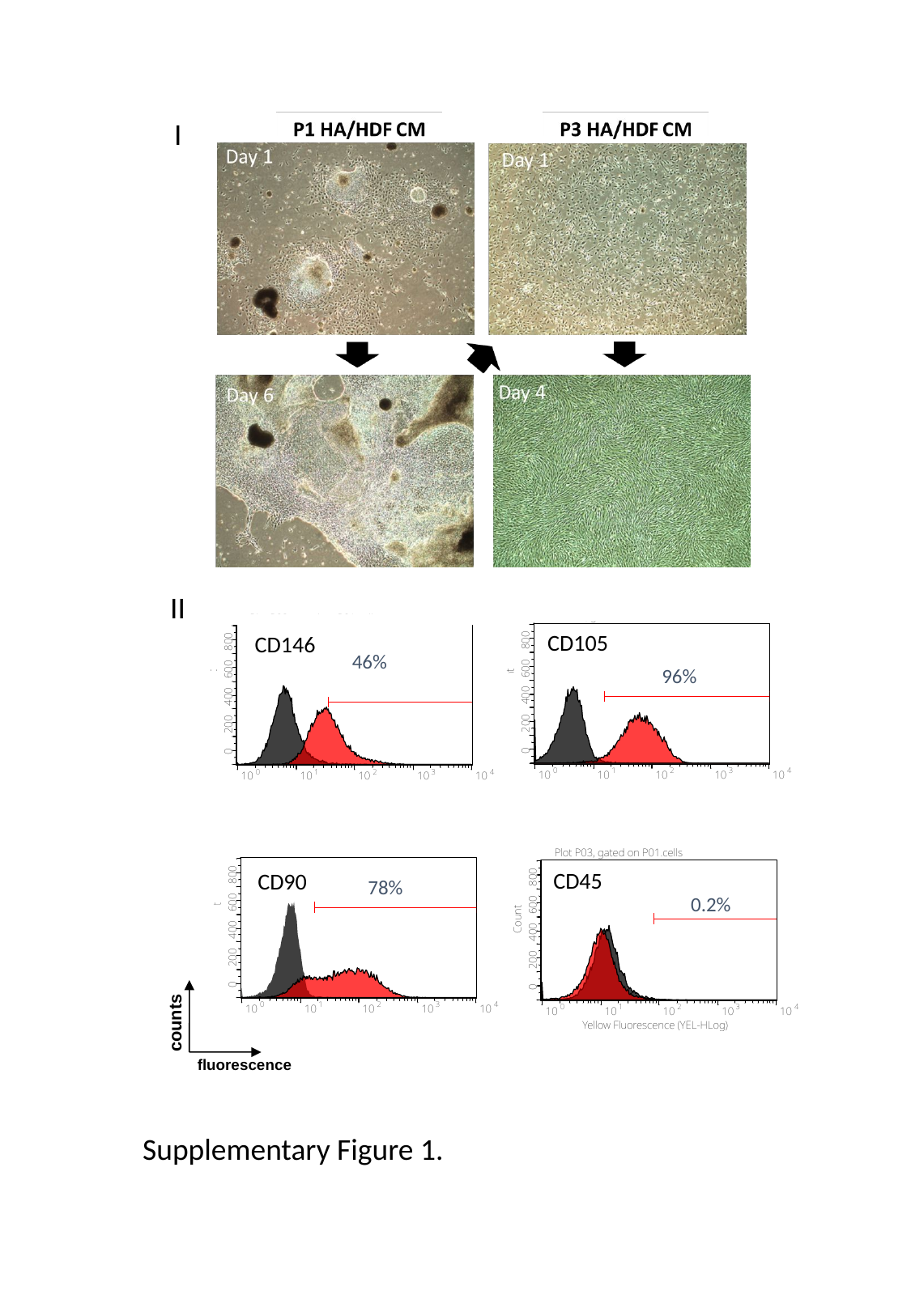

I
CD146, 46%
CD105
CD146
46%
96%
CD90, 78%
CD45
CD90
78%
0.2%
counts
fluorescence
II
Supplementary Figure 1.

## Slide 3
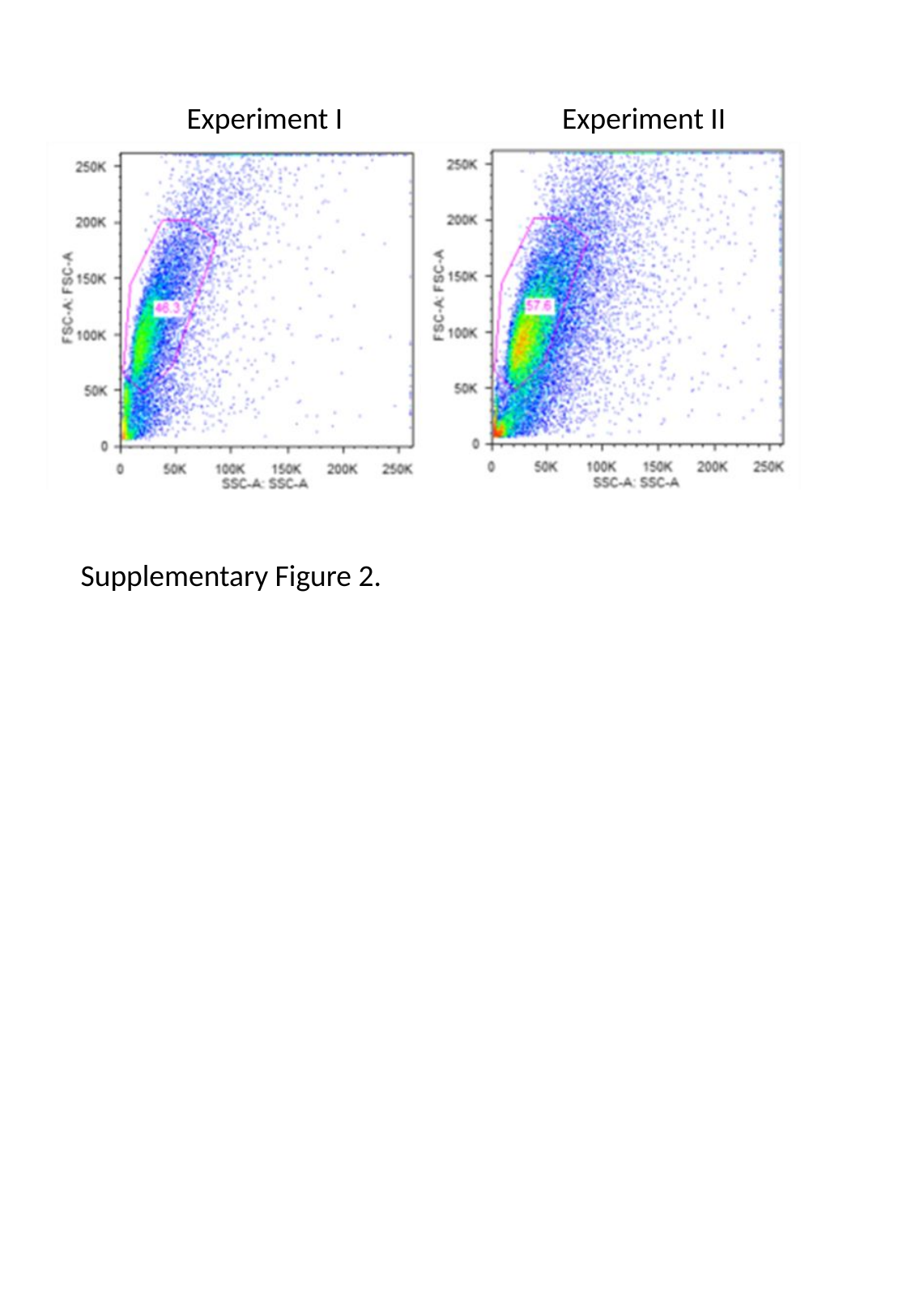

Experiment I
Experiment II
Supplementary Figure 2.

## Slide 4
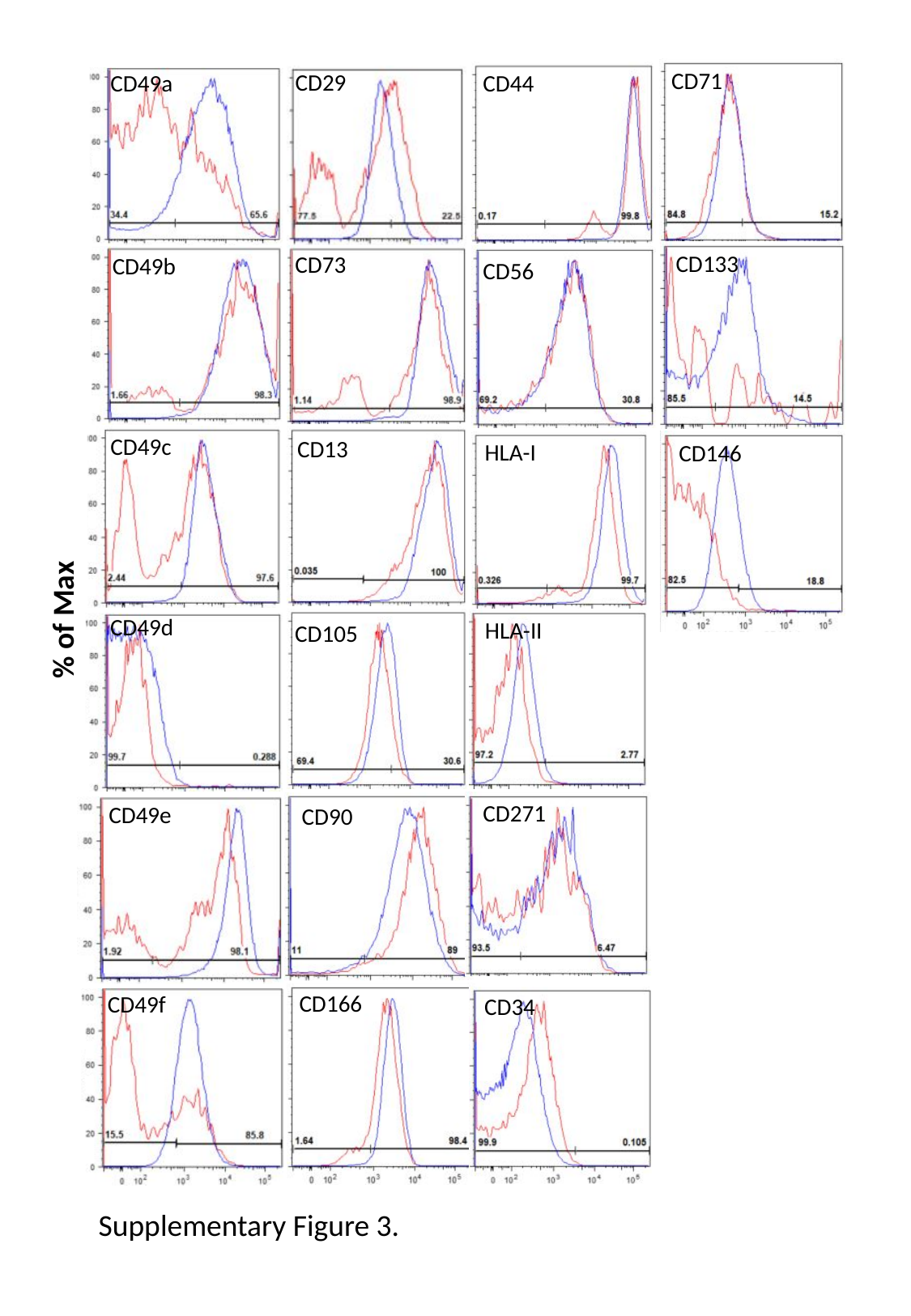

CD71
CD29
CD44
CD49a
CD133
CD73
CD49b
CD56
CD49c
CD13
HLA-I
CD146
CD49d
HLA-II
CD105
CD271
CD49e
CD90
CD166
CD49f
CD34
% of Max
Supplementary Figure 3.

## Slide 5
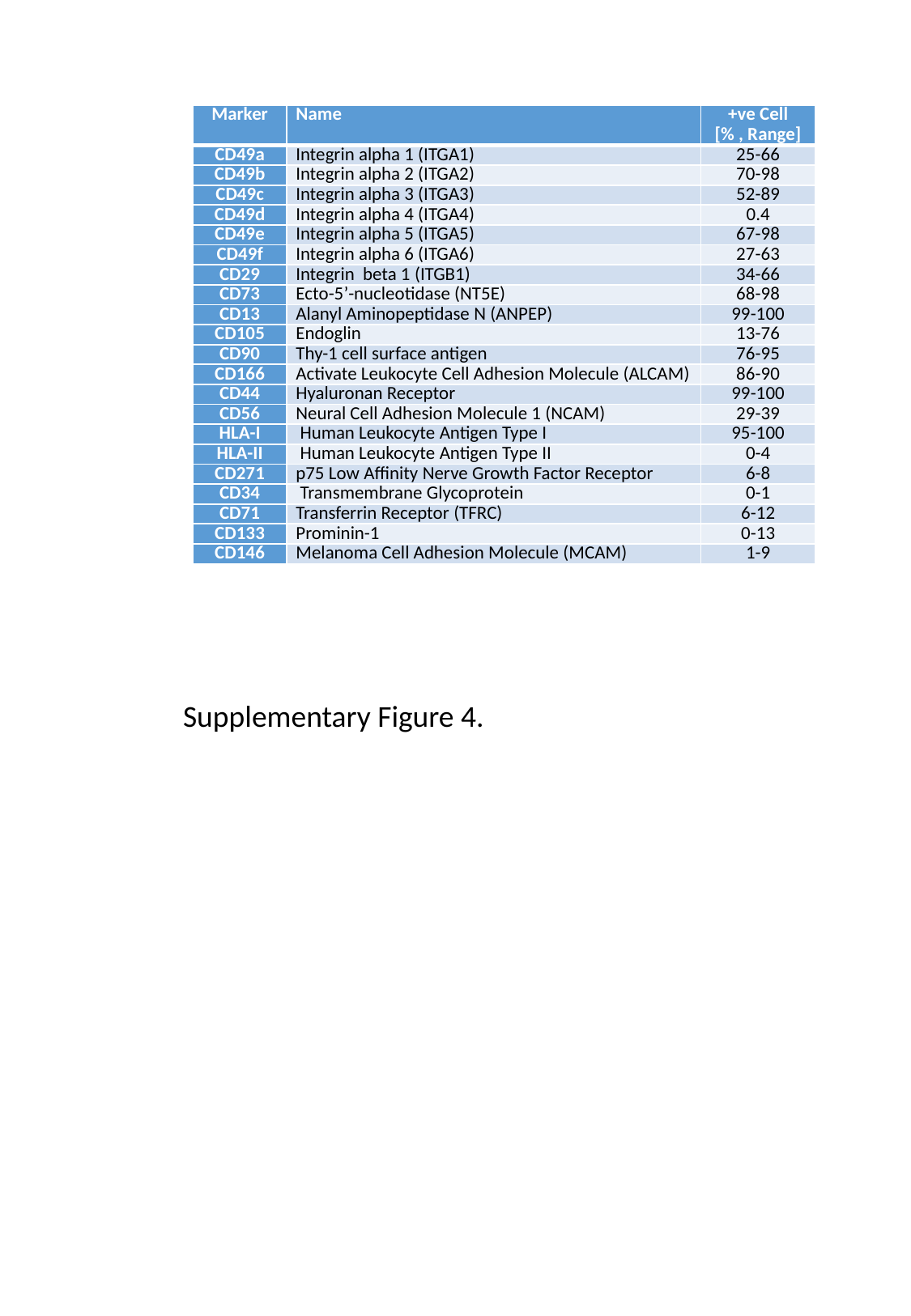

| Marker | Name | +ve Cell [% , Range] |
| --- | --- | --- |
| CD49a | Integrin alpha 1 (ITGA1) | 25-66 |
| CD49b | Integrin alpha 2 (ITGA2) | 70-98 |
| CD49c | Integrin alpha 3 (ITGA3) | 52-89 |
| CD49d | Integrin alpha 4 (ITGA4) | 0.4 |
| CD49e | Integrin alpha 5 (ITGA5) | 67-98 |
| CD49f | Integrin alpha 6 (ITGA6) | 27-63 |
| CD29 | Integrin beta 1 (ITGB1) | 34-66 |
| CD73 | Ecto-5’-nucleotidase (NT5E) | 68-98 |
| CD13 | Alanyl Aminopeptidase N (ANPEP) | 99-100 |
| CD105 | Endoglin | 13-76 |
| CD90 | Thy-1 cell surface antigen | 76-95 |
| CD166 | Activate Leukocyte Cell Adhesion Molecule (ALCAM) | 86-90 |
| CD44 | Hyaluronan Receptor | 99-100 |
| CD56 | Neural Cell Adhesion Molecule 1 (NCAM) | 29-39 |
| HLA-I | Human Leukocyte Antigen Type I | 95-100 |
| HLA-II | Human Leukocyte Antigen Type II | 0-4 |
| CD271 | p75 Low Affinity Nerve Growth Factor Receptor | 6-8 |
| CD34 | Transmembrane Glycoprotein | 0-1 |
| CD71 | Transferrin Receptor (TFRC) | 6-12 |
| CD133 | Prominin-1 | 0-13 |
| CD146 | Melanoma Cell Adhesion Molecule (MCAM) | 1-9 |
Supplementary Figure 4.

## Slide 6
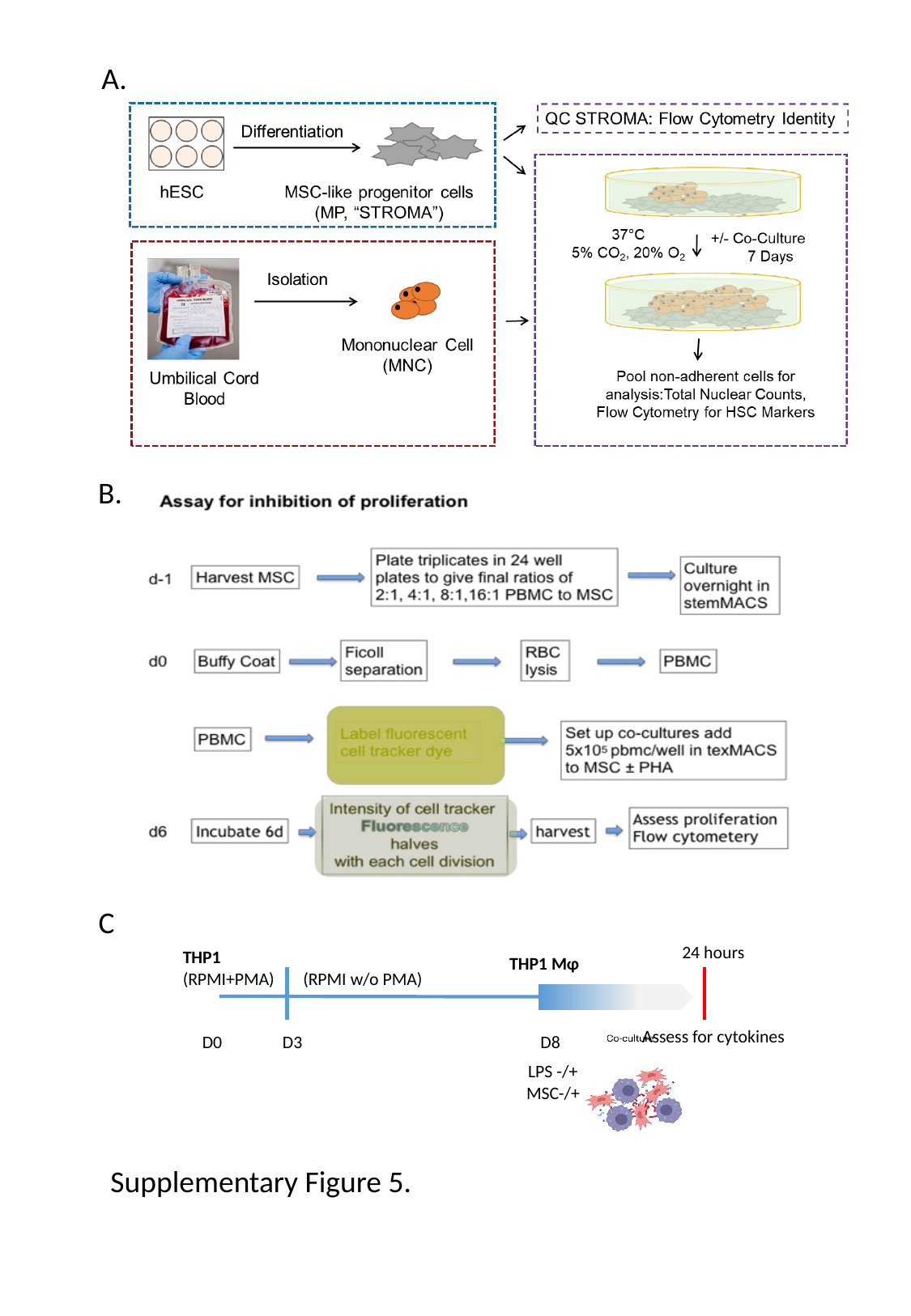

A.
B.
C
24 hours
THP1
(RPMI+PMA)
THP1 Mɸ
(RPMI w/o PMA)
Assess for cytokines
D0 D3 D8
LPS -/+
MSC-/+
Supplementary Figure 5.

## Slide 7
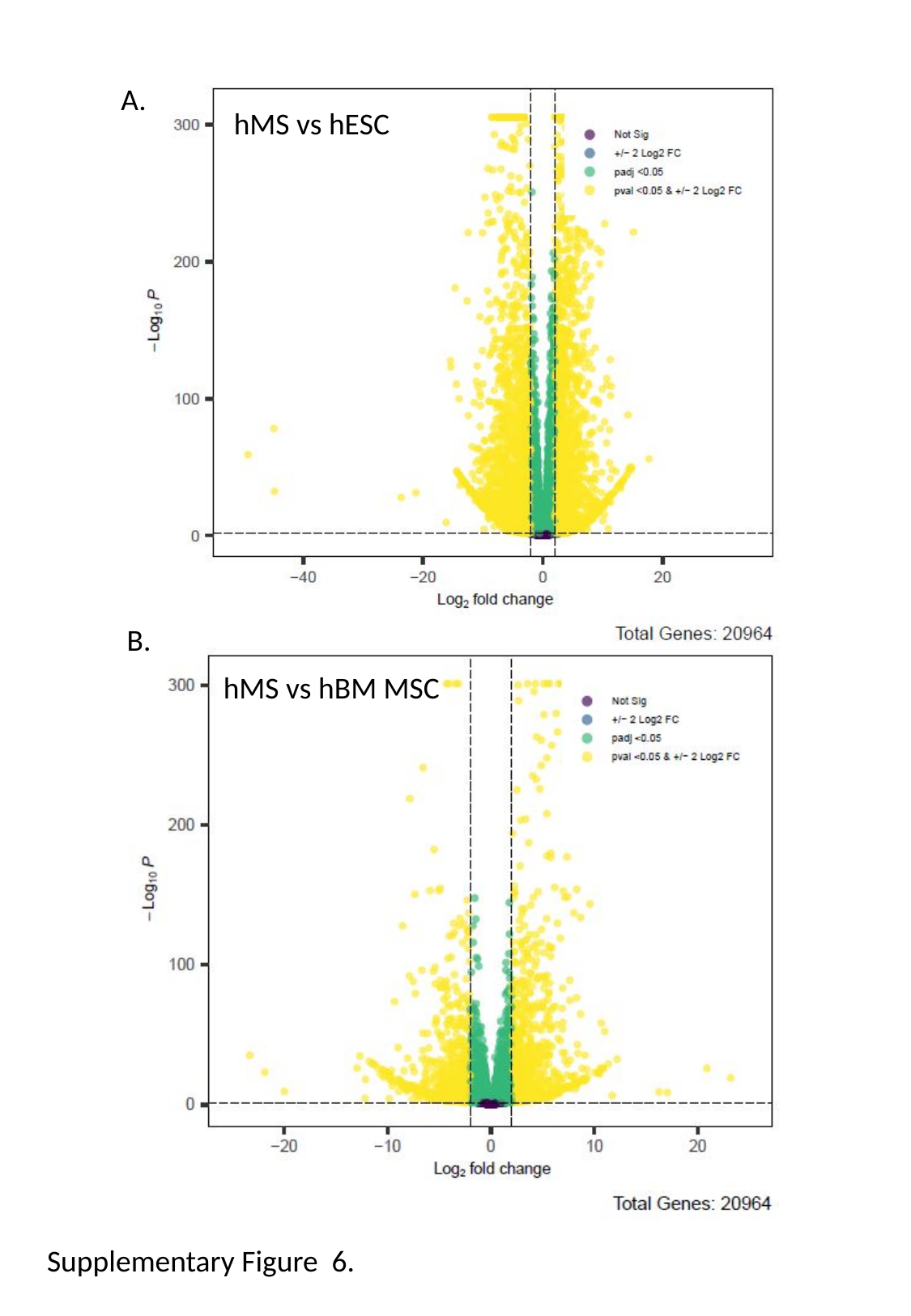

A.
hMS vs hESC
B.
hMS vs hBM MSC
Supplementary Figure 6.

## Slide 8
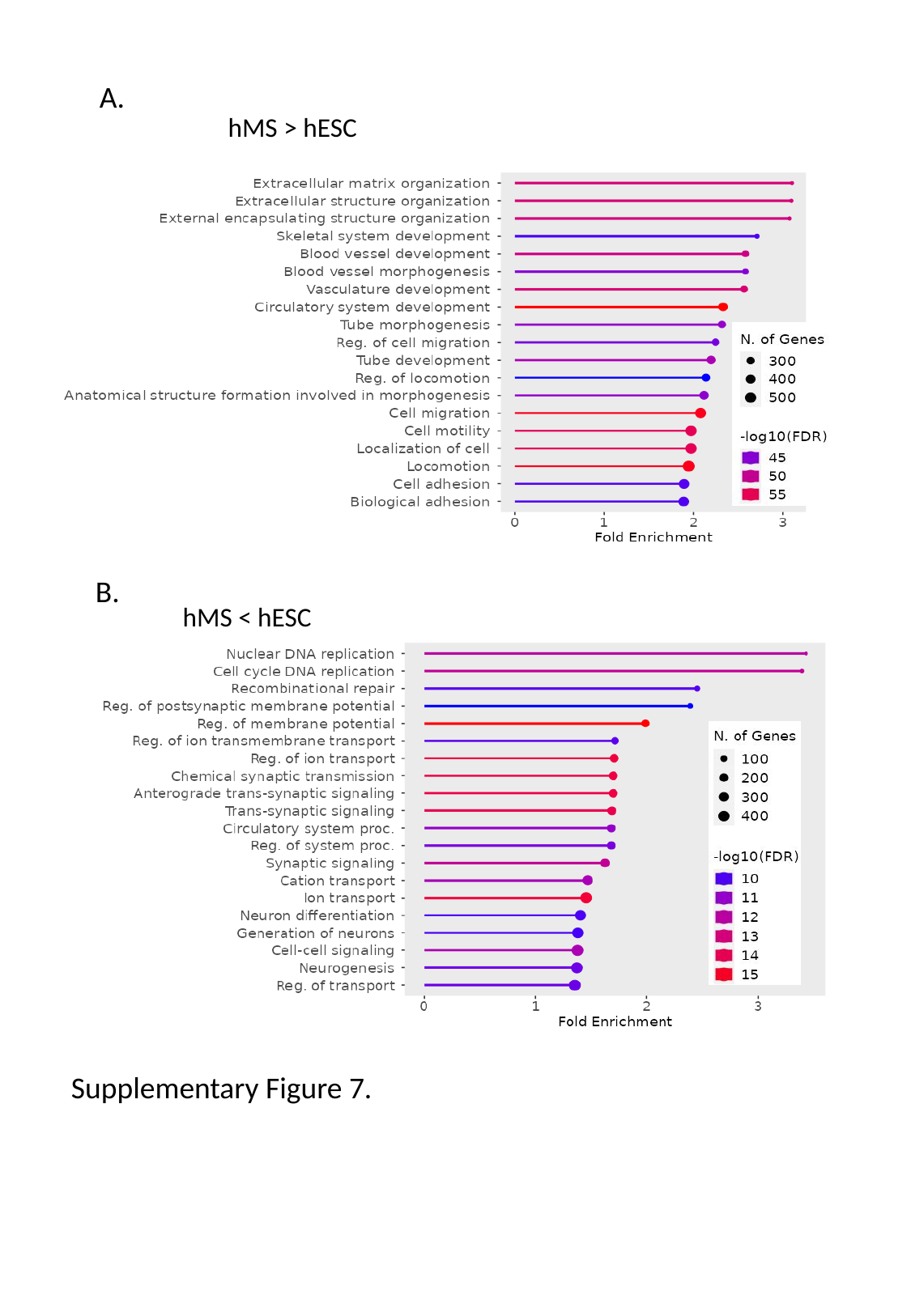

A.
hMS > hESC
B.
hMS < hESC
Supplementary Figure 7.

## Slide 9
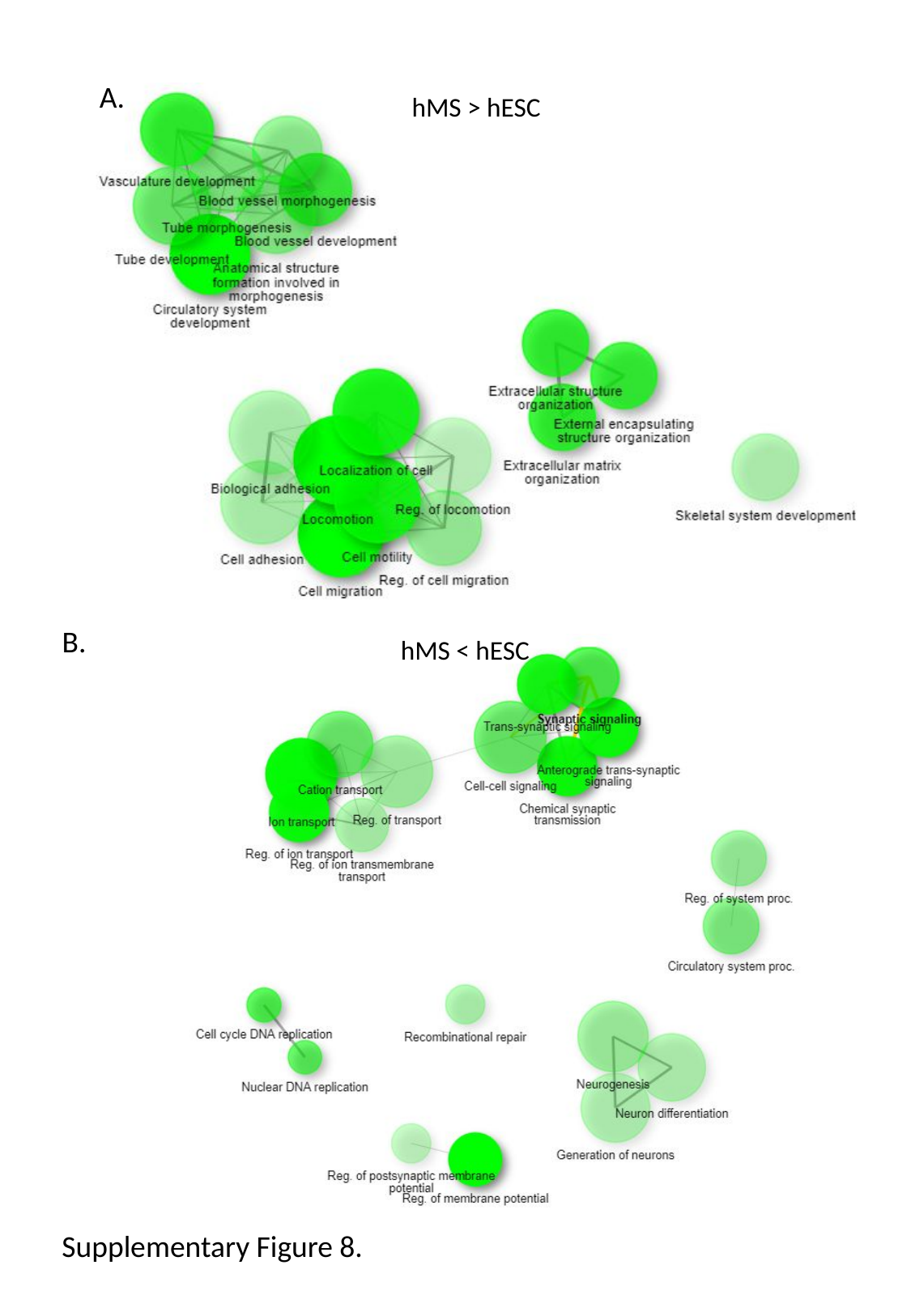

A.
hMS > hESC
B.
hMS < hESC
Supplementary Figure 8.

## Slide 10
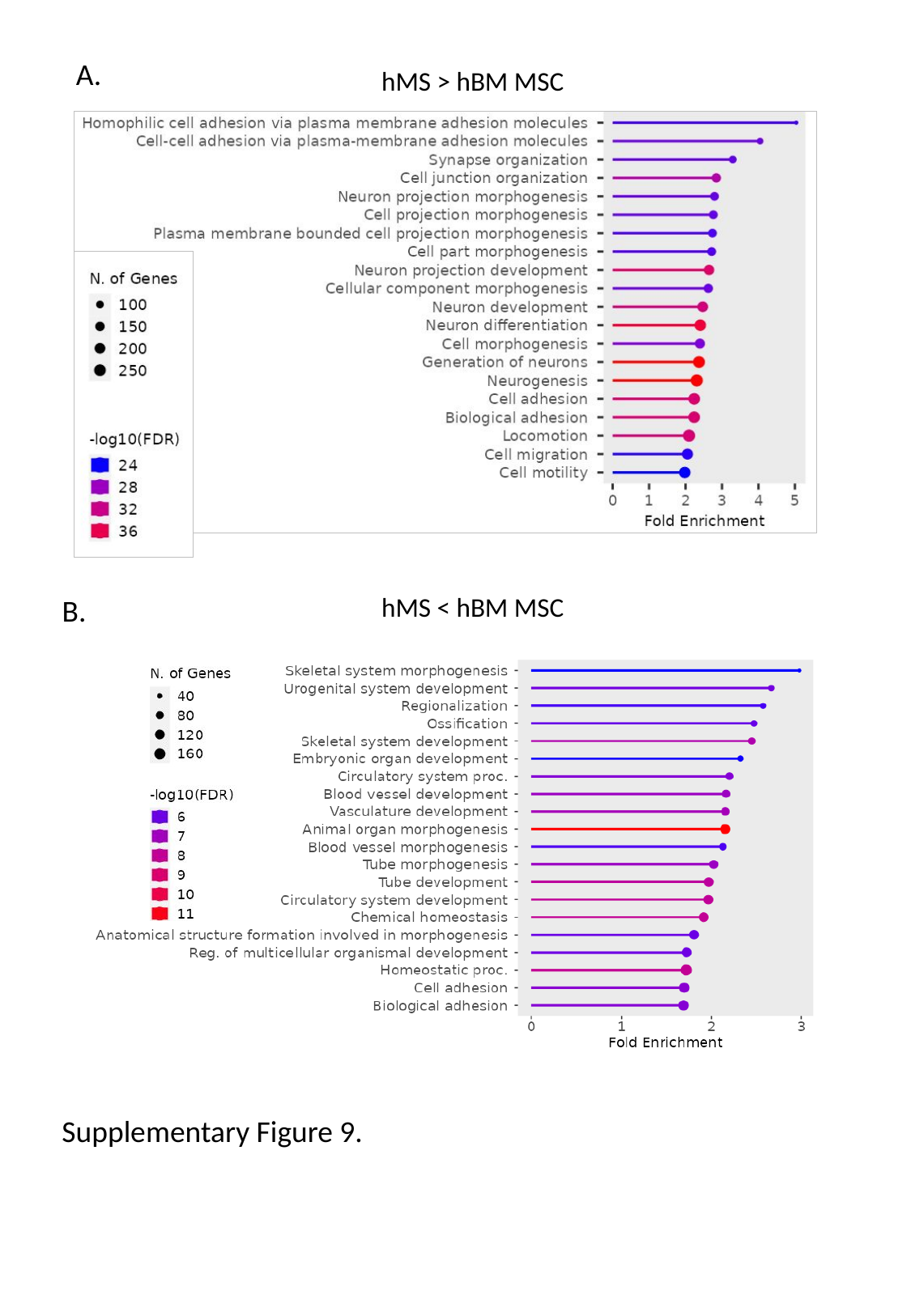

A.
hMS > hBM MSC
hMS < hBM MSC
B.
Supplementary Figure 9.

## Slide 11
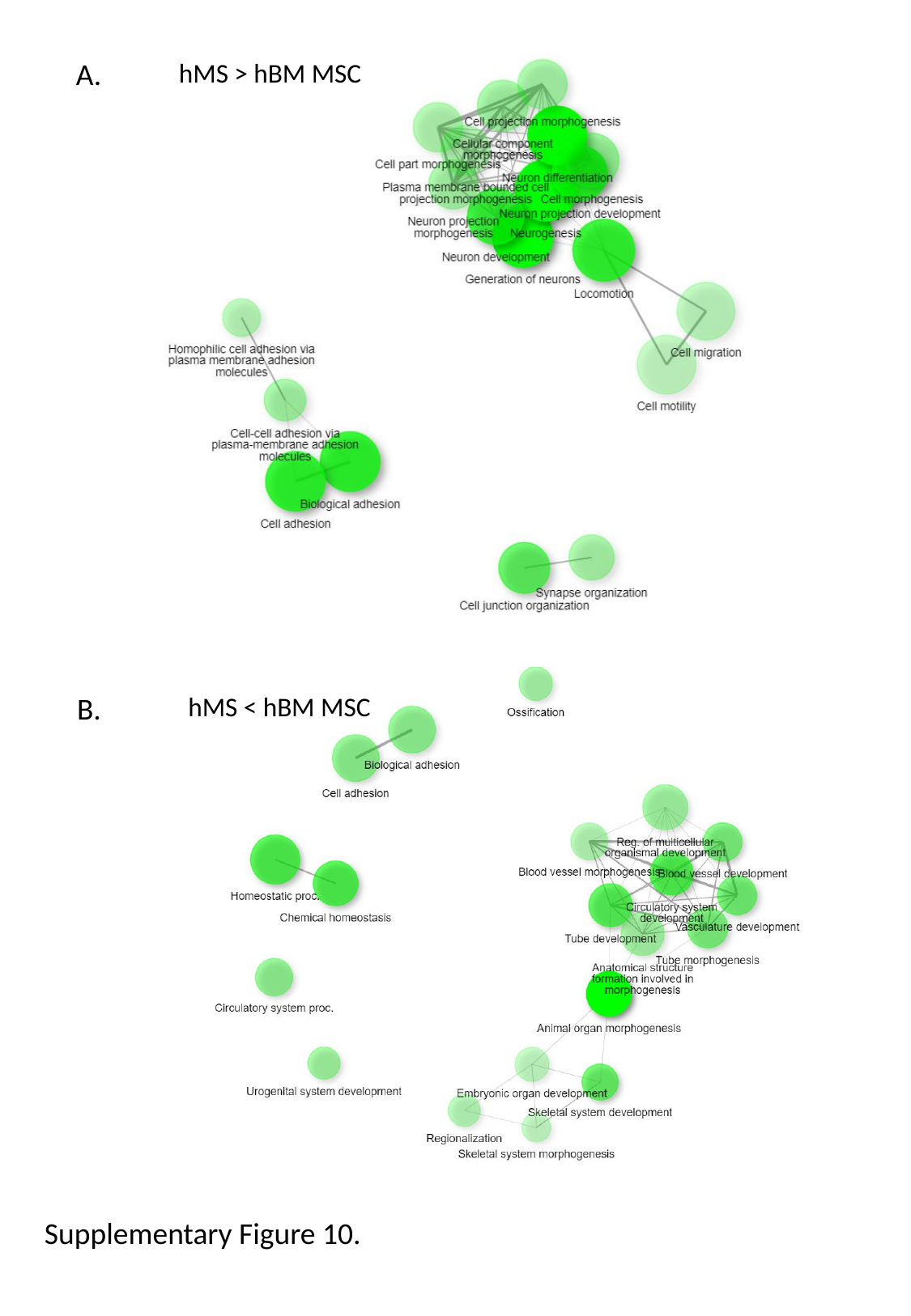

A.
hMS > hBM MSC
B.
hMS < hBM MSC
Supplementary Figure 10.

## Slide 12
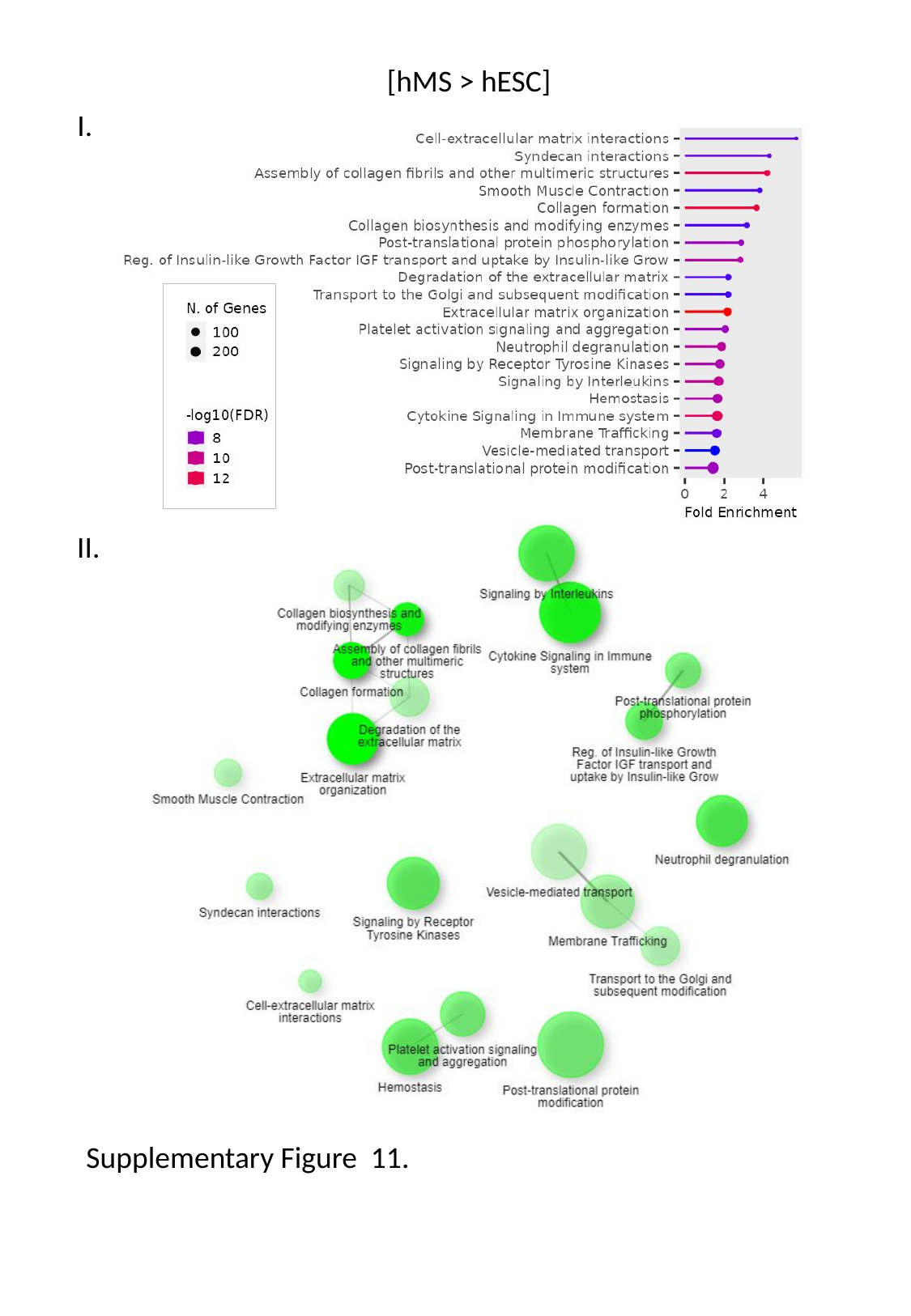

[hMS > hESC]
I.
II.
Supplementary Figure 11.

## Slide 13
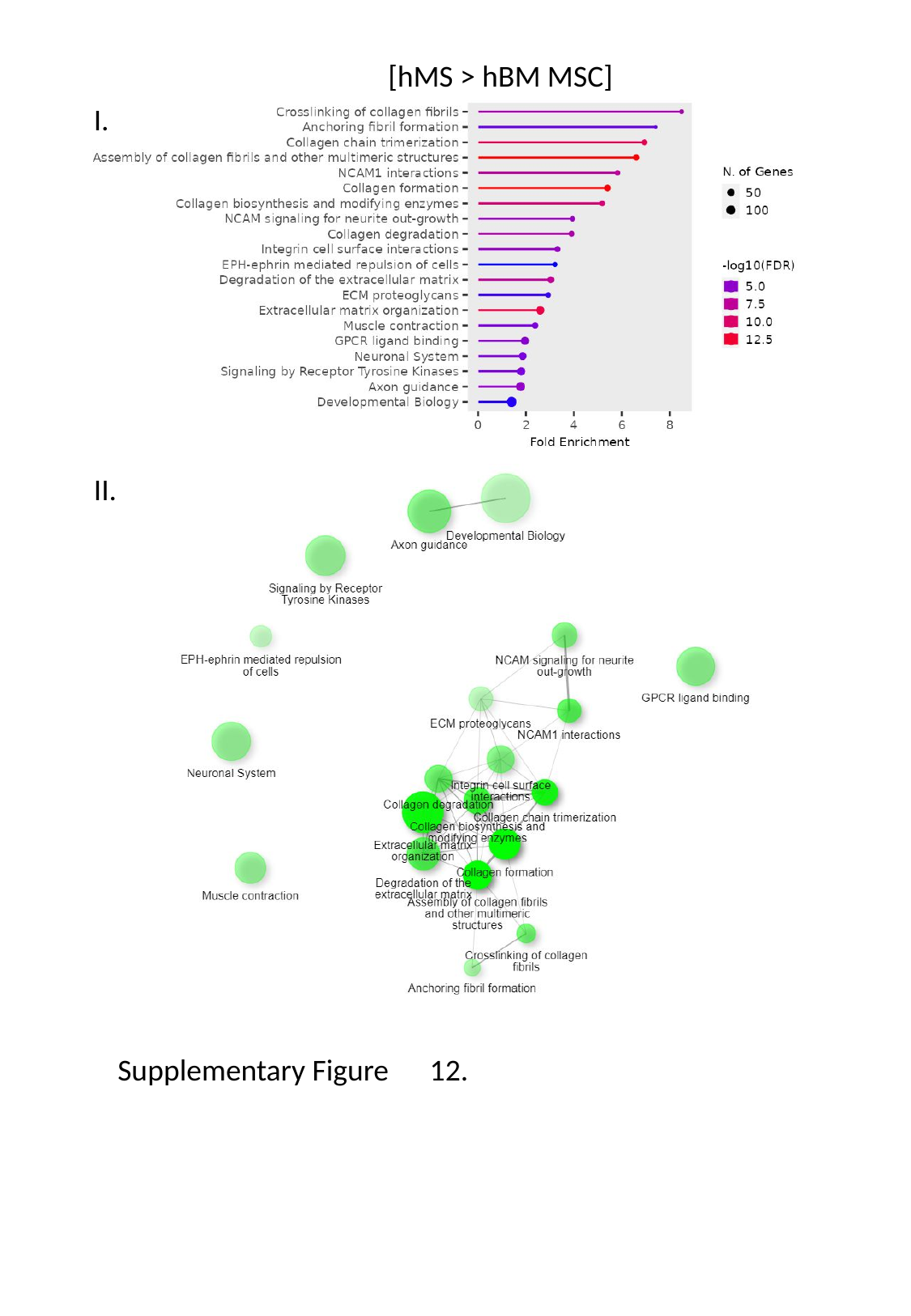

[hMS > hBM MSC]
I.
II.
Supplementary Figure 12.

## Slide 14
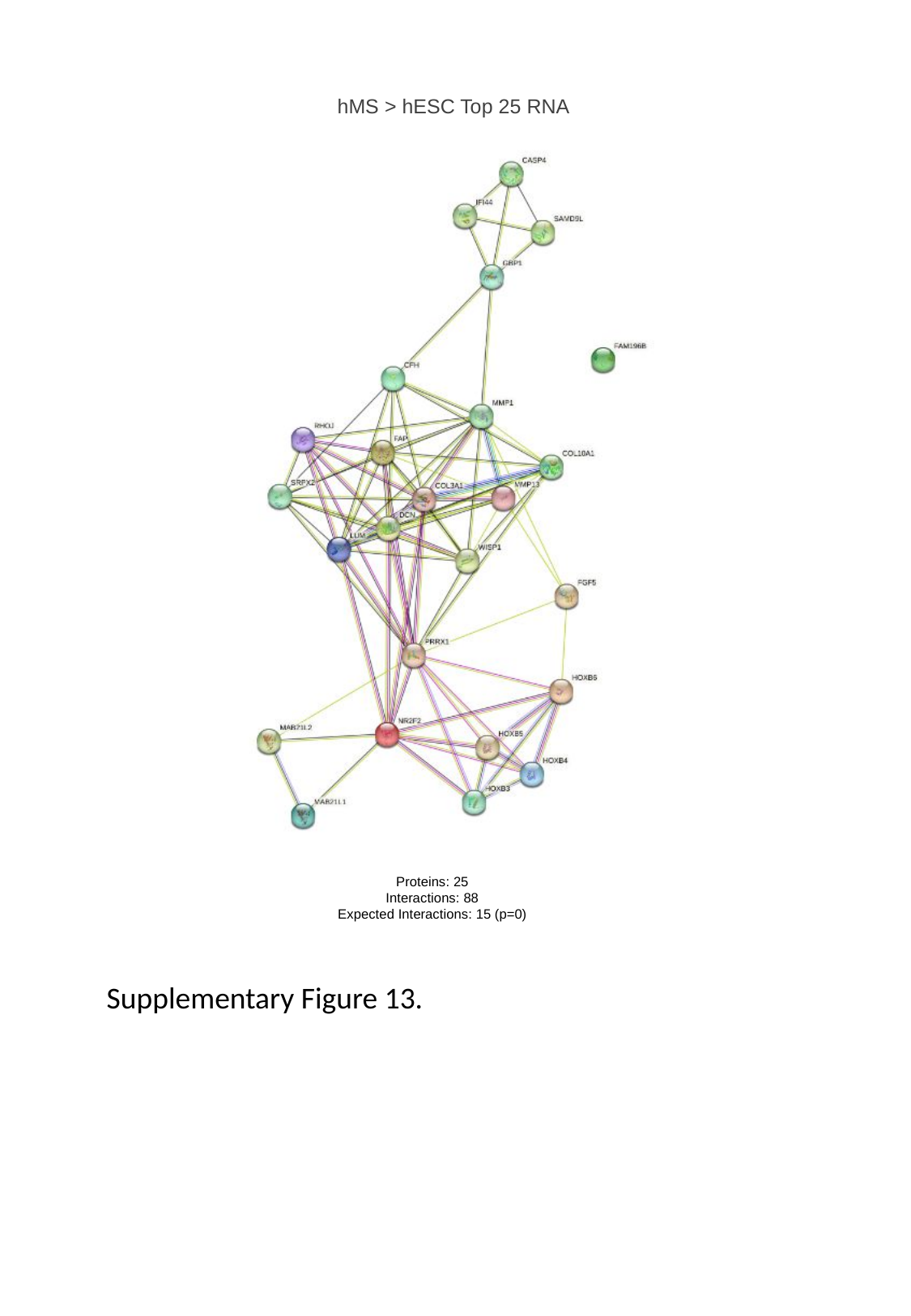

hMS > hESC Top 25 RNA
Proteins: 25
Interactions: 88
Expected Interactions: 15 (p=0)
Supplementary Figure 13.

## Slide 15
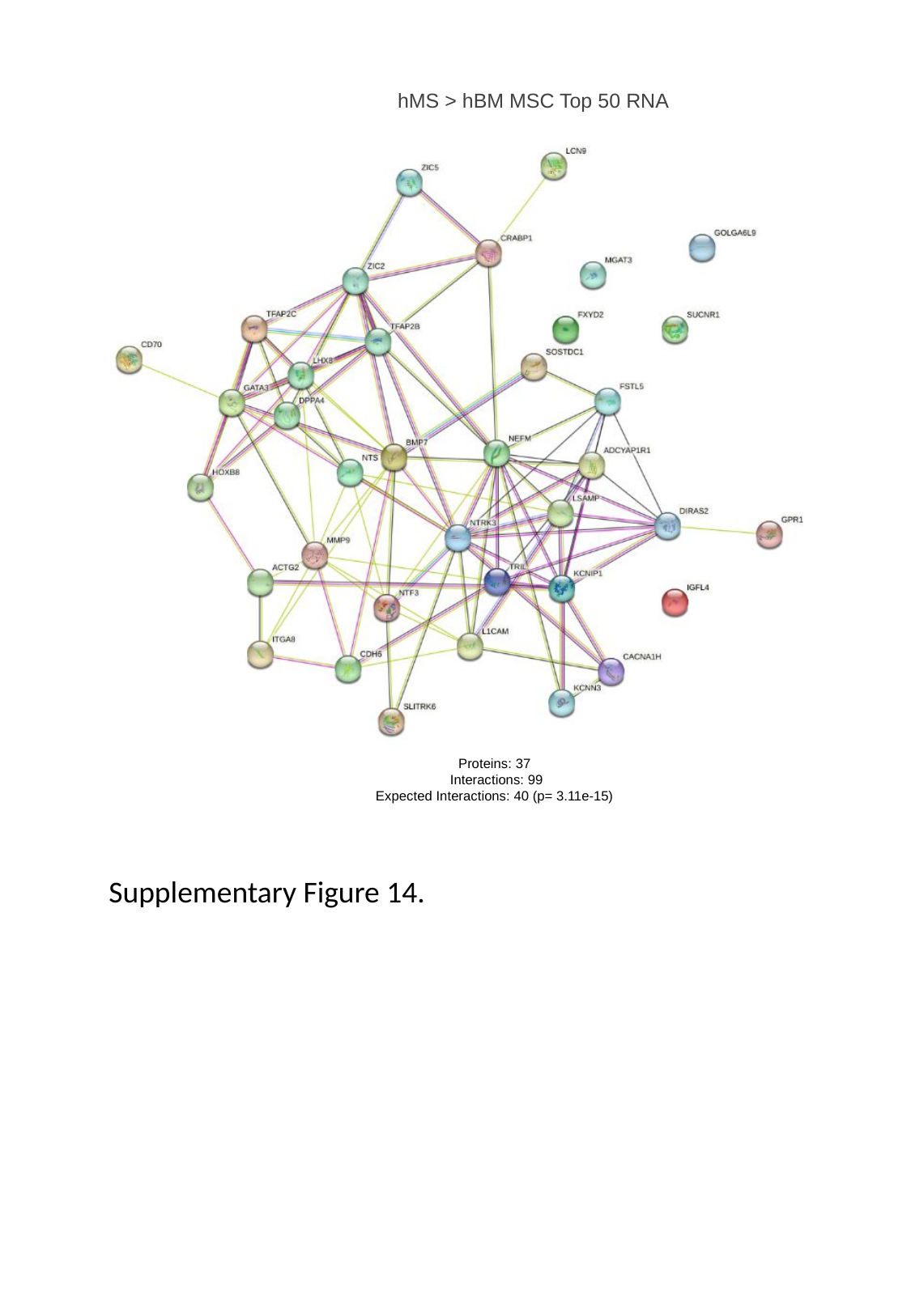

hMS > hBM MSC Top 50 RNA
Proteins: 37
Interactions: 99
Expected Interactions: 40 (p= 3.11e-15)
Supplementary Figure 14.
